# Octavinyl-POSS as a Modifier of Thermoplastic Polyolefin Elastomers Based on Ethylene-Propylene-Diene and Ethylene-1-Octene EPDM/POEs Blends

**DOI:** 10.3390/ma19194216

**Published:** 2026-10-03

**Authors:** Magdalena Lipińska, Patrycja Marek, Monika Gałęziewska

**Affiliations:** Institute of Polymer and Dye Technology, Lodz University of Technology, Stefanowskiego 16, 90-537 Lodz, Poland; 247959@edu.p.lodz.pl (P.M.); monika.galeziewska@p.lodz.pl (M.G.)

**Keywords:** ethylene-propylene-diene/ethylene-1-octene EPDM/POE blends, polyhedral oligomeric silsesquioxanes octavinyl-POSS, viscoelastic properties, thermo-responsive shape-memory behavior

## Abstract

Thermoplastic polyolefin elastomers (TPOs) were developed from ethylene-propylene-diene (EPDM) rubber and ethylene-1-octene copolymers (POEs) differing in melt viscosity and crystallinity. EPDM/POE blends were prepared at a fixed composition by a melt-mixing process readily applicable to industrial manufacturing. To enhance interfacial interactions and network formation, the blends were modified with octavinyl-polyhedral oligomeric silsesquioxane (POSS) and subsequently crosslinked using dicumyl peroxide (DCP). The rheological behavior of the uncured blends was strongly influenced by the viscosity of the POE phase. POSS incorporation, together with peroxide curing, improved viscoelastic response, as evidenced by Cole–Cole analysis, and promoted curing, resulting in higher cure rate index CRI values and shorter optimum cure times τ_90_. The introduction of the POE phase increased the storage modulus G′ of the blends, indicating a reinforcing effect. Furthermore, curing and POSS modification enhanced the tensile properties, leading to a pronounced increase in tensile strength and an approximately fourfold improvement in elongation at break. Crosslinking in the presence of POSS reduced the crystallinity of the POE phase and altered the viscoelastic response by restricting polymer chain mobility, thereby reducing stress relaxation and improving creep-recovery performance. Enhanced interfacial interactions were further confirmed by glass transition behavior. As a result, the EPDM/POE/POSS systems exhibited thermo-responsive shape-memory performance, characterized by higher shape fixity F and shape recovery RR ratios.

## 1. Introduction

Thermoplastic elastomers (TPEs) are a class of polymeric materials composed of more than one phase, and they can be fabricated by blending a semicrystalline thermoplastic polymer with rubber [1]. The mechanical mixing of polyolefins and rubber is the main route for fabricating TPE blends. Thermoset elastomers are more difficult to process, and to reach optimum mechanical performance, they need to be cured. Hence, the creation of thermoplastic elastomers TPE is a useful method to fabricate multiphase materials, being a bridge between rubber and thermoplastic polymers. Thermoplastic elastomers exhibit unique properties: ease of processing, the possibility of being recycled, elasticity and mechanical strength [1].

Mechanically mixed polyolefin thermoplastic polymers with various rubbers are called thermoplastic polyolefin elastomers (TPO/TPE-O) and are a main group that belongs to TPEs. Among them are thermoplastic vulcanizates (TPVs) that consist of isotactic polypropylene (PP) mixed with ethylene-propylene-diene terpolymer (EPDM) [2] or ethylene-propylene rubber (EPR) [3]. Further dynamic vulcanization of these blends can be carried out [1]. Polyolefin elastomers (POEs), such as ethylene-octene and products of copolymerization of olefins, are used in blending with both thermoplastic polymers [4,5,6,7] and rubbers [8,9]. Thermoplastic polyolefin elastomers (TPOs) based on ethylene-octene copolymers and polypropylene can be processed using typical methods, such as injection molding [4]. The presence of POE affects the impact strength of foamed and solid PP/POE blends [4]. Similarly, the impact on the toughness of polyamide 6 (PA6) was observed after mixing with maleic anhydride-grafted ethylene-1-octene [5,6]. The authors of [6] demonstrated that adjustment of octene content in POE together with the molecular weight of ethylene-octene copolymer led to the precise tailoring of impact resistance.

Rheological analysis proved that the structure of the POE component, the octene mer content and the molecular weight of the copolymer were factors affecting the storage E’ and loss modulus E’’ at elevated temperatures due to the various mobilities of polymeric chains [6]. Dynamically vulcanized blends of ethylene-propylene-diene and ethylene-1-octene (EPDM/POE) with various formulations and ratios of both components were investigated [8]. The authors of [8] show that the content of EPDM tailored mechanical enhancement; the tensile strength and tearing strength reached a maximum for 20 wt% of EPDM. Moreover, the storage modulus E’ of dynamically vulcanized EPDM/POE increased to a level higher than that determined for neat POE but was reduced as the EPDM content was increased. Other authors [9] show that for ethylene-propylene-diene and ethylene-1-octene blends, a high degree of compatibility is observed due to the formation of sufficient physical entanglement between both phases. Further, the impact of EPDM content on stress relaxation was determined. In our studies, we applied the Engage^TM^ copolymers as a component of ethylene-propylene-diene and ethylene-1-octene EPDM/POE blends. These copolymers have varied viscoelastic properties due to their chain architecture, octene content and molecular weight. The differences in viscosity of the melted POE component, together with its ability to form a crystalline phase, can be factors influencing both the mixing of ethylene-1-octene with ethylene-propylene-diene rubber and the morphology of the TPO blend and viscoelastic properties in the melted and solid states of the final material.

The morphology control of elastic rubber blends is a key issue in programming final mechanical properties [10]; thus, various strategies are applied to enhance compatibility of blend components. Compatibilization strategies include adding block or graft copolymers or reactive polymers and adding low-molecular compounds or selective crosslinking agents that can enhance the physical or chemical bonds between phases [11]. Among them, compatibilization promoted by incorporation of solid micro- or nanoparticles can be an effective method [7,11]. Introduction of fillers into polymers is attractive from an economical and industrial point of view because fillers can be added directly during processing or mixing of blend components. Fillers with similar affinity to both phases can be localized at the interfacial region, leading to the stabilization of blend morphology [11]. It was shown that fillers with a higher aspect ratio can effectively reduce the size of dispersed domains [12]. The application of fillers is a cheap alternative to chemical modification, such as the introduction of grafted chemical groups [5,6]. Deeb et al. [5] demonstrated the impact of modification of ethylene-1-octene using maleic anhydride on the improved blend compatibilization of polyamide 6/maleic anhydride-grafted ethylene-1-octene copolymers. Maani et al. [7] analyzed the viscoelastic properties and morphological stability of the polypropylene and ethylene-1-octene blends under shear flow in the presence of nanosilica. The silica nanoparticles improved morphological stability; the effect was more pronounced when nanoparticles were localized in the ethylene-1-octene phase. To favor the localization of silica nanoparticles, the authors of [7] additionally modified the polypropylene phase by grafting maleic anhydride. In particular, fillers can be used not only as compatibilizers but also as nucleating agents, increasing the degree of crystallization, leading to the fabrication of material with better mechanical and thermal properties [12].

In our studies, polyhedral oligomeric silsesquioxanes (POSS) with functional octavinyl groups were chosen as compatibilizing nanoparticles for TPO blends based on ethylene-propylene-diene rubber and ethylene-1-octene copolymer. Polyhedral oligomeric silsesquioxanes are nanometer-sized organic/inorganic hybrid materials with an empirical formula of (RSiO_1.5_)_n_, where n = 8, 10, 12. In POSS particles, the inert inorganic cage built of -Si-O- bonds has organic side groups with various reactive/nonreactive functionalities [13]. The type of the side groups is a crucial factor controlling the reactivity of POSS when incorporated into various polymeric materials. The reactive groups, together with the inorganic POSS structure, can provide molecular reinforcement, increase thermal stability, and enhance filler/polymer compatibility [13]. POSS particles with various hydrophilic/hydrophobic groups were used to improve superhydrophilicity/superhydrophobicity of coatings [14]. Epoxy-polyhedral oligomeric silsesquioxanes were used to modify the strength, toughness, and relaxation of nanocomposite epoxy vitrimers [15]. The furan ring-grafted POSS particles together with liquid polysulfide rubber can be applied as toughening modifiers, leading to enhanced mechanical and thermal properties of epoxy resins [16]. Other authors [17] proved that intermolecular interactions such as van der Waals forces, hydrogen bonds, and CH-π interactions are formed between functional phenyl or aminophenyl groups of POSS particles and silicon rubber, resulting in better mechanical properties and thermal stability. Octamaleamic acid-POSS (OM-POSS) was applied as the adhesion enhancer in silica-reinforced silicone rubber; moreover, the addition of OM-POSS enhanced the thermal stability of cured rubber [13]. Similarly, Chen et al. [18] observed a synergistic effect of divinyl-hexa[(trimethoxysilyl)ethyl]-POSS used as a crosslinker and fumed silica on the thermal and thermo-oxidative stability and mechanical properties of room temperature vulcanized (RTV) silicone rubber.

Recent developments demonstrate that functionalized POSS derivatives can serve as multifunctional additives in various elastomer matrices, acting, for example, as environmentally sustainable crosslinking agents, compatibilizers, and reinforcing fillers. Konnola et al. [19] prepared a hybrid organic/inorganic elastic composite based on carboxyl-terminated polyacrylonitrile-butadiene rubber using glycidyl polyhedral oligomeric silsesquioxane as a crosslinker; the formation of chemical bonds between rubber and POSS epoxy groups was confirmed using the FTIR method. The use of fillers as crosslinking agents for elastomers is promising due to the possibility of incorporating inorganic particles into the network. This approach allows for the minimization of filler aggregation, and the presence of the filler also exerts a reinforcing effect. It is an environmentally friendly approach that allows a decrease in the amount of crosslinking agents or accelerators, which are often environmentally harmful. Peng et al. [20] have developed an environmentally friendly strategy for crosslinking ethylene-vinyl acetate rubber using NH_2_-POSS, which allows avoiding the use of peroxides that can generate harmful substances during decomposition. They found [20] that NH_2_-POSS effectively crosslinked the rubber and further contributed to improving the dispersion of silica introduced into the system, resulting in a composite with enhanced tensile strength. Yi et al. [21] reported that octa(3,3,3-trifluoropropyl) polyhedral oligomeric silsesquioxanes retarded natural rubber curing but enhanced network integrity. Despite promoting the formation of a denser physical network, confirmed by reduced swelling, 8F-POSS increased fractional free volume, improving interfacial frictional slippage, leading to a synergistic “reinforcing-dissipation” effect [21]. Natural rubber vulcanizates modified by 8F-POSS exhibited better damping properties [21]. Modified POSS nanoparticles can also be used to impart unique special properties, such as increased thermal conductivity, to elastomer nanocomposites [22]. To achieve this, Mirizzi et al. [22] synthesized hybrid modified Al_2_O_3_/POSS nanoparticles and used them as a filler for polybutadiene.

In our work we propose the application of octavinyl-POSS as a nanoparticles able to be incorporated into ethylene-propylene-diene and ethylene-1-octene blend. The presence of eight vinyl groups, which are active in peroxide curing, should lead to the promotion of network curing even at low concentrations of peroxide and, as a result, has an impact on the mechanical and viscoelastic properties of EPDM/POE. Octavinyl-POSS particles were used by other authors as additives to natural [23] and silicone rubber [24]. The formation of chemical bonds between octavinyl-POSS and natural rubber was confirmed, leading to enhancement of mechanical properties of cured NR rubber [23]. In our previous studies [25], we applied octavinyl-POSS as an additive to ethylene-propylene rubber EPR and hydrogenated acrylonitrile-butadiene EPR/HNBR blends. The curing analysis demonstrates that octavinyl-POSS, due to the ability of vinyl groups to participate in peroxide curing, reduced the optimal cure time and enhanced the cure rate. Furthermore, the activation energy E_a_ of curing was also decreased. Based on our previous studies [25], showing the effect of POSS on the crosslinking of blends, we decided to use these nanoparticles to modify EPDM/POE blends. Application of octavinyl-POSS should reduce the amount of peroxide used to cure the EPDM/POE blend.

Thermoplastic polyolefin elastomers such as ethylene-1-octene copolymers are applied in various industries, e.g., automotive, bumper covers, air dams, body side telecommunication, wires and cables, medical, roofing. Unfortunately, in some areas of application, the permanent set limits their use [1]. Dynamic crosslinking of EPDM/POE blend using dicumyl peroxide was previously studied by other authors [26]. Crosslink nodes hinder the mobility of POE chains, reducing the degree of crystallinity; smaller crystals were formed, but curing also enhanced compatibility of both phases [26]. The authors of [26] proved that crosslinking occurred in both phases and also at the interphase.

EPDM/POE blends can be applied as temperature-programmed shape-memory polymers (SMPs) [9]. SMPs are materials that can rapidly change previously programmed temporary shape into their original shape under temperature stimulation [27]. Thermal-sensitive SMPs usually have a physical or chemical crosslinking structure and a crystalline/amorphous hard phase. Melting temperature T_m_ can be used as a programing/switch temperature. Chatterjee T. et al. [9] demonstrated that shape of EPDM and Engage^TM^ 8440 blends can be programmed at 60 °C temperature. Blends with a higher ratio of EPDM showed better shape fixity and shape recovery behavior [9].

In our opinion, the modification of EPDM/POE blends and its shape-memory behavior can be improved by using POSS particles able to participate in curing. Furthermore, the application of POSS should also influence the viscoelastic behavior of EPDM/POE blends. Although POSS nanoparticles have been widely investigated as functional nanofillers in polyolefin- and elastomer-based materials, previous studies have mainly focused on their effects on morphology, mechanical properties, thermal stability, and rheological behavior. In contrast, limited attention has been paid to EPDM/POE systems.

The aim of this study was to investigate the influence of the type of ethylene-1-octene copolymer on the morphology, crosslinking behavior, mechanical performance, viscoelastic response, thermal properties, and shape-memory characteristics of EPDM/POE blends synergistically modified with octavinyl-POSS and dicumyl peroxide (DCP). Three commercially available POE grades differing in their physical characteristics were incorporated into EPDM-based systems and subsequently characterized using rheological, mechanical, thermal, morphological, and shape-memory analyses. Particular attention was paid to identifying the most promising composition capable of combining good mechanical performance with efficient thermally activated shape-memory behavior. The obtained results provide insight into the relationship between blend composition and the resulting functional properties of EPDM/POE/DCP/POSS materials. To the best of our knowledge, no study has systematically evaluated the simultaneous influence of DCP and octavinyl-POSS on curing characteristics, viscoelastic response, and shape-memory performance in EPDM/POE blends. Overall, this work demonstrated that the synergistic modification with DCP/octavinyl-POSS can be applied to adjust the mechanical performance of EPDM/POE blends.

## 2. Materials and Methods

### 2.1. Materials and Preparation of EPDM/POE and EPDM/POE/POSS Blends

EPDM/POE blends were formulated using Keltan^®^ 4450S EPDM rubber supplied by LANXESS Buna GmbH, Marl, Germany. The elastomer exhibited a Mooney viscosity of 42 MU (ML 1 + 4 at 125 °C), an ethylene content of 52 wt.%, and an ethylidene norbornene ENB content of 4.3 wt.% [28]. As the thermoplastic phase, three commercially available Engage™ ethylene-1-octene copolymers (POEs) were selected (Dow Inc., New York, NY, USA), further denoted as 8137, 8452, and 8180. For all selected POE copolymers, comparable glass transition temperatures (about −55 °C) and different melting temperatures of their crystalline phase (in the range of 47–66 °C) are reported [29]. The selected POEs differed in Mooney viscosity, MU (ML 1 + 4 at 121 °C): 8137 4 MU; 8452 11 MU; and 8180 37 MU. According to the producent [29], the total crystallinity was in the range 13–20%.

Consequently, all EPDM/POE blends were compounded in a micromixer (Brabender 146 PlastiCorder, Brabender GmbH, Duisburg, Germany) at temperatures exceeding 100 °C to ensure complete melting of the POE phase throughout the mixing process.

**Figure 1:**
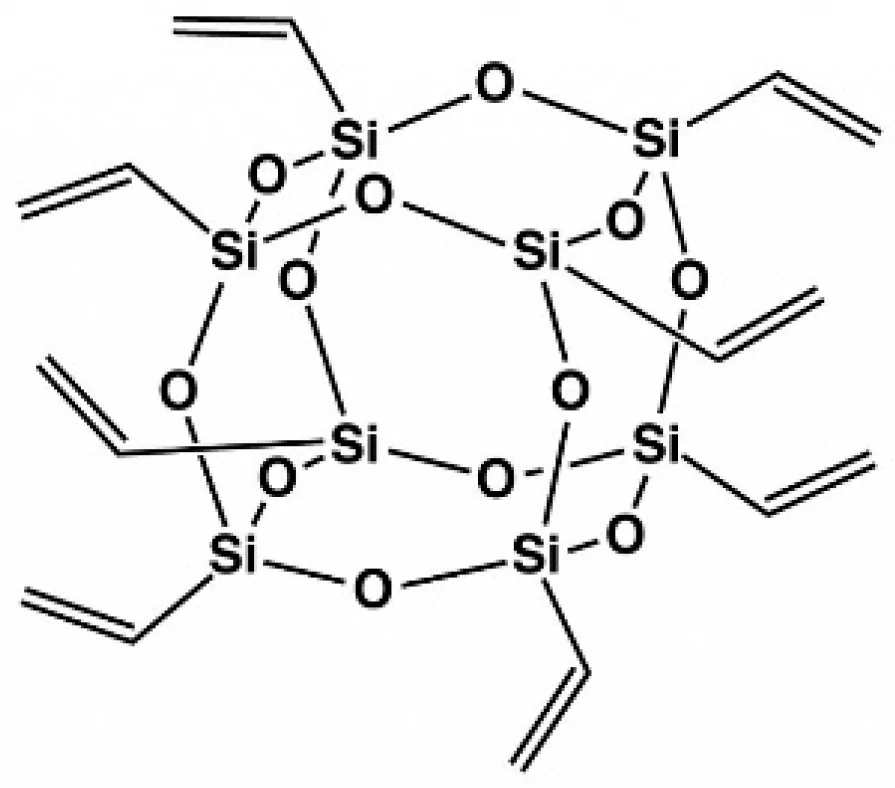
Structure of octavinyl-POSS.

Octavinyl-polyhedral oligomeric silsesquioxane, further denoted as octavinyl-POSS (Hybrid 131 Plastics, USA, AM0265, Hattiesburg, MS, USA), was employed as a nanostructured modifier for the EPDM/POE blends. The structure of the POSS is shown in Figure 1.

EPDM/POE blends were compounded in a Brabender internal mixer (Brabender 146 PlastiCorder, Brabender GmbH, Duisburg, Germany) maintained at 100 °C. The formulations EPDM/POE were prepared at a fixed weight ratio of 80/20 (40 g of EPDM and 10 g of POE). Prior to blending, the POE granules were melted in the mixing chamber for 10 min at 5 rpm. EPDM was then gradually introduced over a period of approximately 1 min at 30 rpm. Following the addition of the elastomer, the rotor speed was increased to 100 rpm and mixing was continued for 10 min to promote intensive distributive and dispersive mixing, resulting in a homogeneous blend morphology.

Functionalized EPDM/POE/POSS blends were prepared using a similar method. Immediately after the addition of EPDM, 0.5 g of octavinyl-POSS was added to the mixture. The addition of EPDM and POSS was completed within approximately 1 min while maintaining the rotor speed at 30 rpm. Following the incorporation of all components, the rotor speed was increased to 100 rpm and mixing was continued for 10 min to promote efficient dispersive and distributive mixing, thereby ensuring homogeneous distribution of the POSS nanoparticles throughout the polymer matrix. In the final stage, the rotor speed was reduced to 50 rpm, and 0.1 g of dicumyl peroxide (DCP) was introduced as a crosslinking initiator. Mixing was then continued for an additional 2 min to achieve preliminary dispersion of the peroxide within the blend while minimizing the risk of premature crosslinking.

### 2.2. Methods

#### 2.2.1. Rheological Tests at 100 °C

As part of the study, various types of rheological measurements were conducted at a chosen mixing temperature of 100 °C for EPDM/POE blends and EPDM/POE/POSS nanocomposites using a D-RPA 3000 rheometer (MonTech, Columbia City, IN, USA) in accordance with ASTM D6204 [30]. The first test involved determining the dynamic viscosity and complex viscosity at 100 °C and viscoelastic parameters such as storage shear modulus G’, loss shear modulus G″ and tan δ as a function of plasticization time (2 min). Measurements were performed using 0.5 Hz (3.14 rad·s^−1^) and a 0.2% oscillation strain, equivalent to low shear rates (0.08 s^−1^), to study the material properties in the linear viscoelastic range. The mean ± standard deviation values of storage shear modulus G’, loss shear modulus G’’, loss factor tan δ, complex viscosity η*, and dynamic viscosity η’ were calculated based on five independent tests.

The second type of study involved analyzing dynamic viscosity as a function of shear rates in the range of 0–315 s^−1^. Additionally, storage G′ modulus, loss modulus G″ and loss factor tan δ were determined at 100 °C as a function of applied frequency in the range of 0.5–300 rad·s^−1^, at a high applied oscillation strain of 100% outside the linear viscoelastic region.

#### 2.2.2. Curing of EPDM/POE/POSS Blends at 160 °C

The effect of POSS and DCP on EPDM/POE crosslinking was studied using a D-RPA 3000 rheometer (MonTech, Columbia City, IN, USA), according to ISO 6502−3:2018 [31]. For all samples, the elastic torque was measured as a function of time (30 min) at a constant frequency of 1.7 ± 0.1 Hz, temperature of 160 °C, and oscillation amplitude of 0.5° (equivalent to 7%). Based on these measurements, the following parameters were determined: minimum and maximum torque (S’_min_ and S’_max_, respectively), induction time t_i_, time of 5% increase in elastic torque S’, TC_5_, optimal curing time τ_90_,. After curing, samples were cooled down in the rheometer to 70 °C and the viscoelastic properties were analyzed. Storage modulus G’, loss modulus G’’, and loss factor tan δ were determined as a function of oscillation strain in the range of 0.1–100% at a frequency of 1 Hz.

#### 2.2.3. Static and Dynamic Mechanical Tests

Flat sheets for tensile and dynamic tests were compression molded using a hydraulic press maintained at 160 °C. The compounded material was placed between preheated mold plates and subjected to the molding process. Prior to curing, the samples were degassed through three evacuation cycles to minimize the presence of entrapped air and volatile species. The materials were then compression molded under a pressure of 200 bar. Upon completion of the molding and crosslinking process, the resulting sheets were removed from the press and allowed to cool to room temperature under ambient conditions. After cooling, the samples were cut for particular tests. Tensile properties were evaluated in accordance with ISO 37:2017 [32] using a universal testing machine (Zwick model 1435, ZwickRoell GmbH, Ulm, Germany). The tests were performed at room temperature with a constant crosshead speed of 500 mm·min^−1^. For each formulation, a minimum of five dumbbell-shaped specimens were tested. Tensile strength at break and elongation at break were determined from the resulting stress–strain curves. The strain at 100% elongation SE_100_, tensile strength TS, and elongation at break E_b_ were calculated as the mean ± standard deviation from five independent measurements.

The shape-memory behavior of partially crosslinked EPDM/POE/POSS blends was evaluated using a single thermomechanical programming and recovery procedure according to Scheme S1 (Supplementary Materials). Three different samples of each composition were used and programmed according to the procedure described below. A rectangular specimen with an initial length l_0_ was first heated in a laboratory oven to the transition temperature T_trans_ of 80 °C for 5 min. The heated sample was mounted and subsequently elongated using a metal frame that allowed it to be stretched to the desired length. After stretching while maintaining the temperature above the transition temperature T_trans_ a length of l_e_ was measured. The samples in the stretched state remained at 80 °C for an additional 5 min. To fix the temporary shape, the elongated specimen was cooled together with the metallic frame in cold water for 5 min while the external load was removed, resulting in a partial contraction of the sample from l_e_ to l_f_. After cooling to a temperature below T_trans_, samples with a programmed stretched shape were left at room temperature for 10 min and the length l_f_ was measured. The unloaded specimen was then reheated to 80 °C for 10 min, causing recovery of the original shape and a corresponding change in length from $l_{f}$ to $l_{r}$.

The shape-memory performance was quantified using two characteristic parameters: shape fixity F and shape recovery ratio RR, calculated according to Equations (1) and (2), respectively [9].

(1) $$F\left(\%\right)=\frac{l_{f}-l_{0}}{l_{e}-l_{0}}\cdot 100$$

(2) $$RR\left(\%\right)=\frac{l_{f}-l_{r}}{l_{f}-l_{0}}\cdot 100$$

where F is the shape fixity ratio, RR is the shape recovery ratio, l_0_ is the initial specimen length measured at 25 °C prior to deformation, l_e_ is the length of the deformed specimen measured at T > T_trans_, l_f_ is the length after shape fixation and unloading at T < T_trans_, and l_r_ is the length after shape recovery upon reheating to T > T_trans_. The shape fixity ratio F and shape recovery ratio RR were calculated as the mean ± standard deviation from three independent measurements.

The shape fixity ratio F reflects the ability of the material to maintain the programmed temporary shape after removal of the external load, whereas the shape recovery ratio RR describes the ability of the material to recover its original shape upon thermal activation. For an ideal shape-memory material, both parameters approach 100%, indicating complete shape fixation and full shape recovery.

Dynamic rheological measurements were performed using an ARES G2 rotational-oscillatory rheometer (TA Instruments, New Castle, DE, USA) using a 25 mm diameter plate-on-plate geometry. The following tests were performed: amplitude sweep test (measurement conditions: oscillation strain range 0.01–100%, constant frequency 10 rad·s^−1^, temperature 20 °C); frequency sweep test (measurement conditions: constant oscillation strain amplitude 0.05%, frequency range 0.1–628 rad·s^−1^, temperature 20 °C); and temperature sweep test to determine the glass transition temperature T_g_ (measurement conditions: constant oscillation strain amplitude 0.1%, constant frequency 1 Hz). The measurement was performed while the sample was heated from −100 °C to 20 °C using a heating speed of 10 °C·min^−1^. The parameters such as storage modulus G’, loss modulus G’’, and loss factor tan δ were determined.

The relaxation and creep behavior of the EPDM/POE blends and EPDM/TPE/POSS nanocomposites was investigated using a TA Instruments Ares G2 oscillatory rheometer fitted with a 25 mm parallel-plate geometry. Stress relaxation tests were conducted under small-amplitude deformation conditions by applying a step strain of 1%. The decay of stress and modulus G was recorded over a period of 300 s at room temperature 20 °C to assess the relaxation dynamics and network characteristics of the investigated materials.

The modulus, $G\left(t\right)$, obtained from the stress relaxation experiments, was fitted using the generalized Maxwell model represented by the Prony series (Equation (3)) [33,34]:

(3) Gt=Ge+∑iGie−tτi

where $G\left(t\right)\;$denotes the modulus at time t, $G_{e}\;$is the equilibrium modulus corresponding to the residual stress remaining after an infinitely long relaxation time, $G_{i}$ are the Prony series coefficients (relaxation moduli), and $\tau_{i}\;$are the characteristic relaxation times associated with the individual terms of the series. Each Prony element represents a distinct relaxation mechanism occurring within the material, while the number of terms, i, corresponds to the number of Maxwell elements used in the generalized Maxwell model. In the present study, Prony series consisting of three terms (i=3) were employed to model the experimental relaxation data. The fitting procedure and Prony series analysis were performed using TRIOS^®^ Software (version 3.1.5.3696, TA Instruments).

The creep-recovery behavior of the investigated materials was evaluated using a two-step protocol. During the creep phase, a constant stress of 100 Pa was applied for 100 s, and the time-dependent strain response was recorded. Subsequently, the stress was removed, initiating the recovery phase, during which strain recovery was monitored for 100 s. The test was used to assess the viscoelastic response and elastic recovery characteristics of the studied materials.

#### 2.2.4. Thermogravimetric Analysis (TGA), Differential Scanning Calorimetry (DSC) and Scanning Optical Microscopy (SEM)

Thermogravimetric analysis (TGA) was performed using a TGA/DSC1 analyzer (Mettler Toledo, Columbus, OH, USA). Samples were heated from 25 to 600 °C under an argon atmosphere and subsequently from 600 to 900 °C in air at a constant heating rate of 10 °C·min^−1^.

The thermal properties of the blends, including the melting temperature (T_m_) and degree of crystallinity, were characterized using a DSC1 differential scanning calorimeter (Mettler Toledo, Columbus, OH, USA). The analyses were carried out over the temperature range from −100 to 150 °C under a nitrogen atmosphere at heating and cooling rates of 10 °C min^−1^. The degree of crystallinity (X_c_) was calculated from both the first and second heating curves according to Equation (4) used by other authors [9,35]

(4) $$\chi_{C}=\frac{\Delta H_{m}}{\Delta H_{c100\%}\cdot W_{i}}\cdot 100\%$$

where χ_C_—degree of crystallinity, ΔH_m_—apparent melting enthalpy of crystallization (J·g^−1^m^−1^), ΔH_c100%_—value of enthalpy of crystallization of 100% crystalline polyethylene, ΔH_c100%_ = 290 J·g^−1^m^−1^ [9,34], and W_i_—weight fraction of polymer in blend.

The morphology of the EPDM/TPE/POSS composites and the dispersion state of the POSS nanoparticles were examined using scanning electron microscopy SEM (Ultra Plus, Carl Zeiss SMT GmbH, Oberkochen, Germany). Samples were cryogenically fractured to obtain representative cross-sectional surfaces. Specifically, strips of the EPDM/POE/POSS composites were immersed in liquid nitrogen for 1 min and subsequently fractured. The resulting fracture surfaces were coated with a thin conductive graphite layer to prevent surface charging during imaging. SEM micrographs were acquired at an accelerating voltage of 3 kV and at various magnifications to evaluate the distribution and dispersion of POSS within the EPDM/POE blend matrix.

## 3. Results

The mechanical behavior of thermoplastic elastomers, including viscoelastic properties and tensile performance, is highly dependent on the volume fraction and morphology of the crystalline phase. Consequently, the variations in crystallinity exhibited by the selected POE grades, together with differences in their viscoelastic properties, are anticipated to significantly influence the mechanical response of the EPDM/POE blend.

To elucidate the effect of POE characteristics on blend performance, a comprehensive rheological and viscoelastic evaluation of the EPDM/POE systems was carried out in both the molten state and at temperatures relevant to their intended applications.

### 3.1. Viscoelastic Properties of EPDM/POE Blends at Processing Temperature 100 °C

The investigated materials consisted of EPDM/POE blends containing various ethylene-1-octene copolymer elastomers (POEs) dispersed within an EPDM rubber matrix, as well as corresponding systems modified with octavinyl-functionalized polyhedral oligomeric silsesquioxane (octavinyl-POSS) nanoparticles.

Due to differences in molecular architecture, the selected Engage™ 8137, 8452, and 8180 copolymers exhibit distinct rheological, thermal, and mechanical properties, which are expected to influence both the processing behavior and the final performance of the EPDM/POE blends. The POE grades were intentionally selected to provide a broad range of melt viscosities, as this parameter plays a critical role in determining the efficiency of melt mixing with EPDM rubber, which possesses a relatively high Mooney viscosity.

Based on the melting characteristics of the selected POEs, a processing temperature of 100 °C was chosen to ensure complete melting of the thermoplastic phase throughout the blending process and to facilitate effective dispersion within the EPDM matrix. To investigate the rheological behavior of the blends under processing-relevant conditions, measurements were conducted at 100 °C using a Rubber Process Analyzer (RPA), a dynamic rheometer widely employed in rubber technology for the characterization of viscoelastic properties during processing and curing [36].

Figures S1–S4 in the Supplementary Materials present the evolution of the viscoelastic parameters as a function of plasticization time at 100 °C. The measurements were conducted at a low shear rate (0.08 s^−1^) to minimize the contribution of shear-induced structural changes and mechanical interactions, thereby ensuring that temperature remained the primary factor governing the rheological response of the investigated materials. It should be underlined that EPDM/POE/POSS blends were studied at 100 °C, a much lower temperature than the curing temperature (160 °C) used further for peroxide-induced crosslinking of EPDM/POE/POSS compositions containing DCP.

Mean values of the viscoelastic parameters determined at 100 °C for the EPDM/POE blends and their POSS-functionalized counterparts are summarized in Table 1.

Regardless of composition, all EPDM/POE blends and their POSS-functionalized counterparts exhibited a decrease in viscoelastic parameters during the first 30 s of testing, indicating melting of the POE phase and progressive plasticization of the EPDM matrix (Figures S1–S4, Supplementary Materials). After approximately 30 s, the measured parameters reached stable values and remained nearly constant throughout the remainder of the experiment. The neat EPDM rubber used in this study was characterized by the highest Mooney viscosity (42 MU at 125 °C). Consequently, the plasticized rubber also exhibited the highest complex viscosity η* at 100 °C (Figure S1, Supplementary Materials). Incorporation of Engage 8137 and Engage 8452 into the EPDM matrix resulted in a reduction in complex viscosity and the storage G′ and loss G″ shear moduli compared with the neat EPDM rubber (Table 1). In contrast, the EPDM/8180 blend exhibited higher values of G’, G″ and complex viscosity η*, indicating enhanced melt elasticity and increased resistance to flow. These results suggest that the rheological response of the blends is strongly dependent on the characteristics of the incorporated POE grade, including its molecular architecture and melt viscosity.

Among the selected POE grades, Engage™ 8180 (Dow Inc., New York, NY, USA) exhibited the highest Mooney viscosity (37 MU at 121 °C) [29], with a viscosity level comparable to that of the EPDM rubber. Similar viscosities of the blend components at the processing temperature are known to play a crucial role in determining the morphology, phase distribution, and overall homogeneity of polymer blends [10,11]. In particular, the viscosity ratio between the constituent polymers influences the size and distribution of the dispersed domains formed during melt mixing. Large differences in the melt viscosities of the blend components may hinder efficient dispersion and promote phase separation, resulting in a less compatible two-phase morphology. In contrast, the closer viscosity match between EPDM and Engage™ 8180 is expected to facilitate more efficient mixing and may improve the homogeneity of the resulting blend. The EPDM/8180 blend exhibited higher storage shear modulus G′ values compared with both the other EPDM/POE blends and the neat EPDM rubber (Table 1). This behavior may be attributed to differences in the molecular architecture of the ethylene-1-octene copolymers. Commercial Engage™ grades differ in molecular weight, molecular weight distribution, and short-chain branching characteristics, all of which affect chain entanglement density and ultimately the rheological response of the material. Consequently, the distinctive molecular structure of Engage™ 8180 contributes to the enhanced viscoelastic properties observed for the corresponding EPDM/POE blend.

Analysis of the storage modulus G′ revealed that the EPDM/8137 and EPDM/8452 blends exhibited relatively low elasticity at 100 °C. From a processing perspective, this behavior is advantageous, as lower melt elasticity reduces the tendency for post-extrusion swell and improves dimensional stability of the extruded product. However, all EPDM/POE blends exhibited loss factor tan δ values below unity under the applied testing conditions (100 °C and a shear rate of 0.08 s^−1^), indicating that the elastic component of the response remained dominant.

The effect of POSS incorporation on the rheological behavior of the plasticized blends was particularly pronounced. The addition of POSS resulted in a reduction in the storage modulus G′ accompanied by an increase in the loss factor tan δ. Simultaneously, a decrease in complex viscosity η* was observed, suggesting that POSS exerted a plasticizing effect on the molten polymer system. This behavior may be attributed to the molecular structure of octavinyl-POSS, which may increase the free volume of the polymer matrix and facilitate chain mobility, thereby reducing resistance to flow. The plasticizing action of POSS nanoparticles has also been reported for other polymer systems and has been observed in our previous studies on molten polymeric materials. Enhanced chain mobility induced by POSS incorporation has frequently been associated with reductions in viscosity and elastic response, particularly at low nanoparticle concentrations where the lubricating effect of the silsesquioxane cages prevails over their reinforcing contribution [37,38].

**Figure 2:**
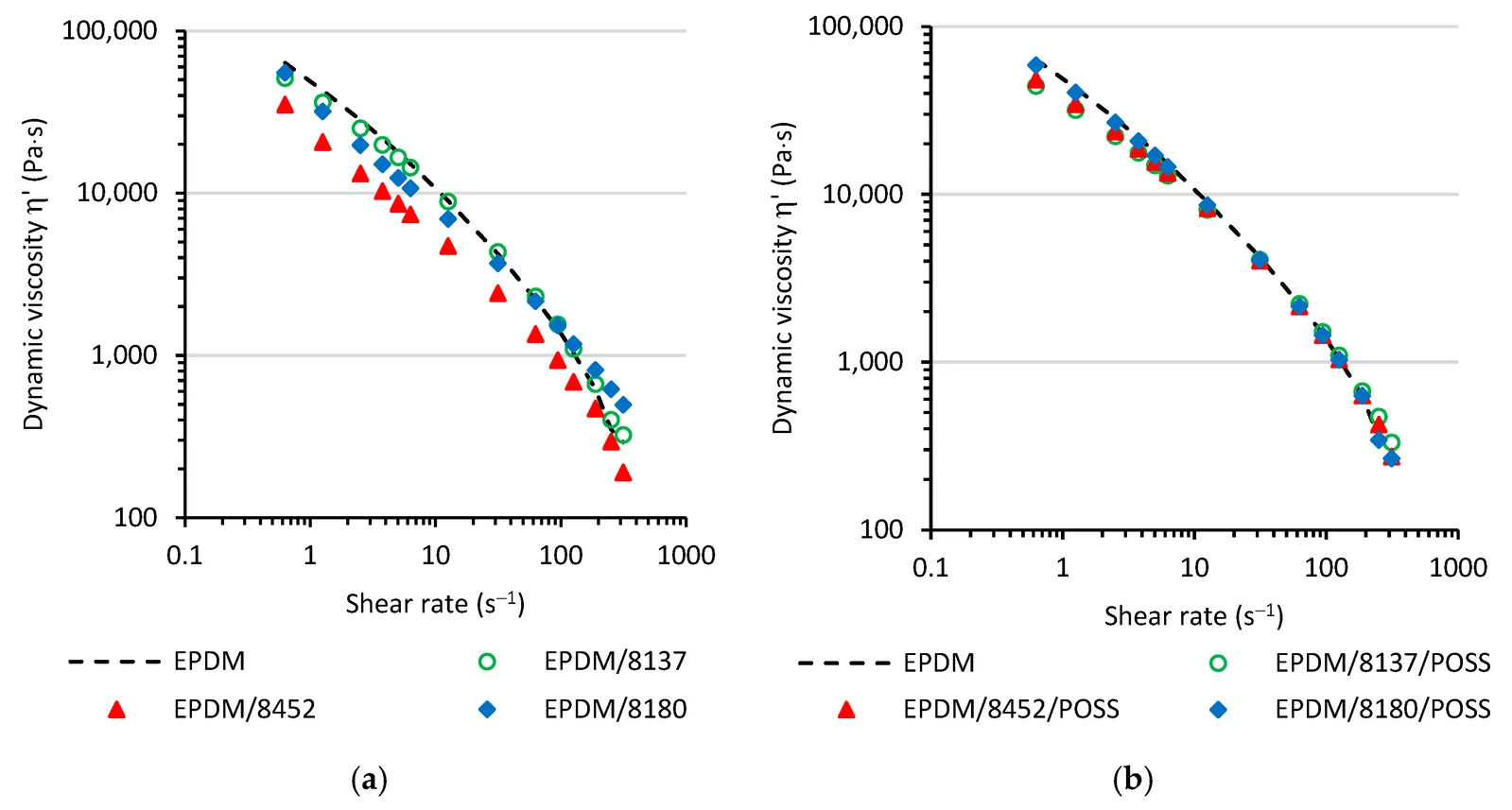
Dynamic viscosity η’ as a function of shear rate (s^−1^) at a processing temperature of 100 °C for EPDM/POE (**a**) and EPDM/POE/POSS blends (**b**).

Since polymeric materials in the melt state are processed at higher shear rates, we determined the viscosity as a function of shear rate (Figure 2). The aim of these measurements was to evaluate the viscous behavior of the resulting EPDM/POE material under conditions corresponding to various processing processes, such as pressing, mixing, and extrusion.

Molten and plasticized polymeric materials typically exhibit non-Newtonian shear-thinning behavior, characterized by a decrease in viscosity with increasing shear rate. The incorporation of POE into the EPDM matrix resulted in a reduction in dynamic viscosity η’ relative to neat EPDM (Figure 2). Furthermore, the EPDM/POE blends displayed more pronounced shear-thinning behavior, which may be attributed to the lower melt viscosity and higher flowability of the POE component. The addition of POSS not only reduced the viscosity of the blends at low shear rates but also contributed to a more favorable rheological response under high-shear processing conditions. In the shear-rate range relevant to industrial melt-processing operations, such as extrusion (>100 s^−1^), the EPDM/POE/POSS systems exhibited viscosity profiles that more closely resembled that of neat EPDM. This behavior suggests that POSS acts as a rheological modifier, improving the processability of the blends before curing, while maintaining sufficient melt elasticity. From a processing standpoint, this effect is advantageous because it promotes more stable flow behavior under high-shear conditions, facilitating material shaping and reducing variations in viscosity during extrusion or other melt-processing operations.

The influence of blend composition on viscoelastic properties under large-amplitude deformation, representative of actual processing conditions, is also of considerable importance. Therefore, the storage modulus G’, loss modulus G’’, and complex viscosity η* were analyzed at 100 °C as a function of oscillation frequency using a strain amplitude of 100%, i.e., outside the linear viscoelastic region. The corresponding results are presented in Figures S5–S7 of the Supplementary Materials. Consistent with the observations obtained under low-deformation conditions, the incorporation of POE led to a reduction in complex viscosity η* across the entire frequency range. The measurements further confirmed the beneficial effect of POSS on the rheological behavior of the EPDM/POE blends. At high frequencies, the complex viscosity of the POSS-functionalized systems approached that of neat EPDM rubber, whereas at low frequencies the modified blends exhibited lower viscosity values, indicating improved processability.

The type of POE employed had a pronounced effect on the viscoelastic response of the blends, particularly on the storage modulus G’. Differences in the melt viscosity of the POE grades may influence blend morphology, affecting both the size and distribution of dispersed POE domains within the EPDM matrix. Consequently, variations in the morphology of the blends give rise to differences in the elastic behavior of the materials at 100 °C.

It should be emphasized that these measurements were performed under a high oscillatory strain of 100%, where both thermal and mechanical effects contribute to the viscoelastic response. Such testing conditions more closely simulate real processing operations, during which materials are simultaneously exposed to elevated temperatures and intensive deformation. Under these conditions, the incorporation of POSS appeared to stabilize the viscoelastic behavior of the blends. The POSS-functionalized materials exhibited a less marked reduction in viscoelastic parameters with increasing deformation and maintained modulus values comparable to those of neat EPDM at high frequencies (Figures S6 and S7, Supplementary Materials). In contrast, at low frequencies, corresponding to longer relaxation times, lower values of the storage modulus G′ were observed, reflecting the influence of both POE and POSS on the relaxation behavior of the blends. These results indicate that the combined effects of blend morphology, interfacial interactions, and POSS incorporation govern the balance between processability and elastic response under processing-relevant conditions.

**Figure 3:**
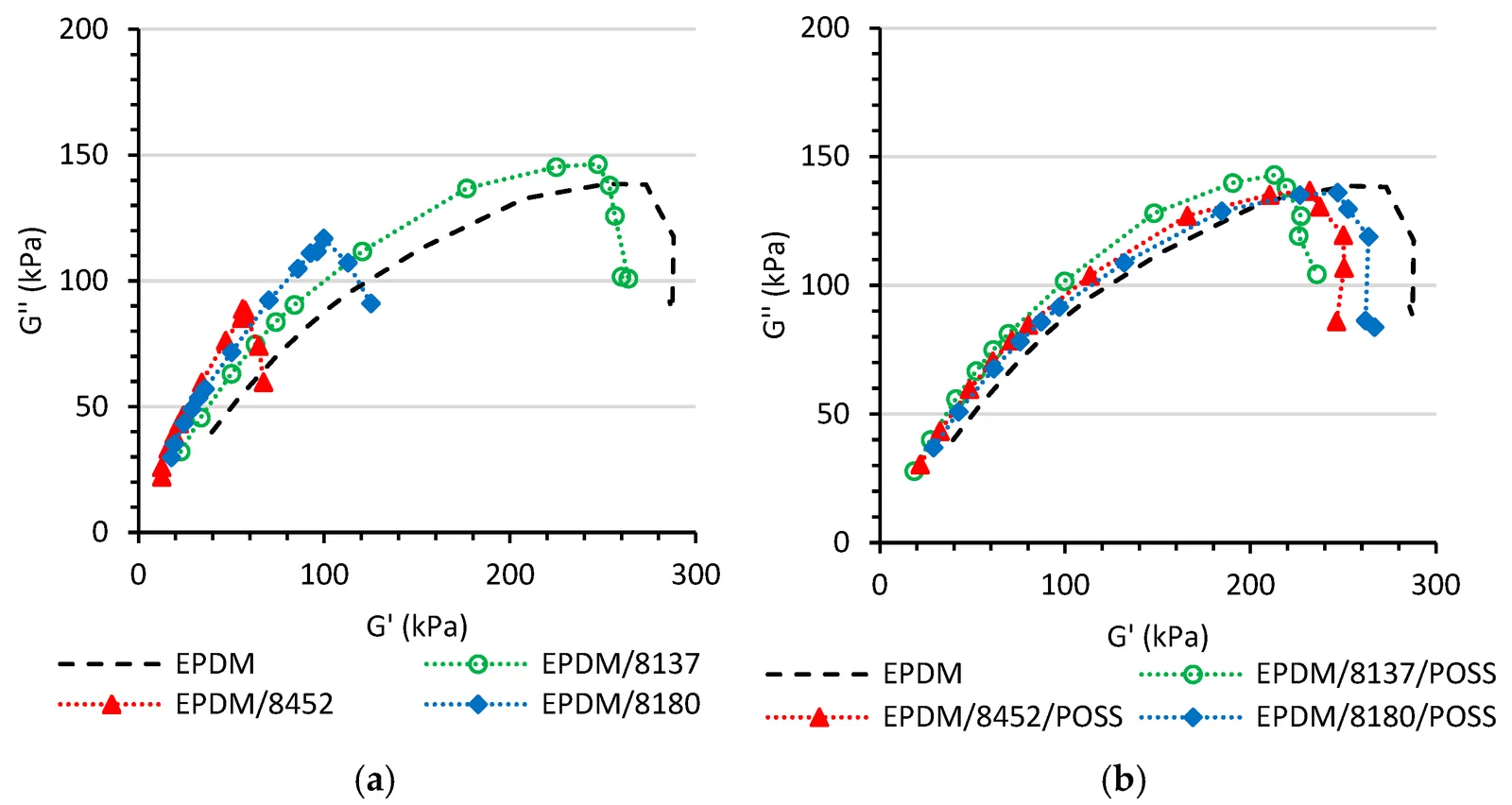
The relationship between storage shear modulus G′ (kPa) and loss shear modulus G″ (kPa) for EPDM/POE (**a**) and EPDM/POE/POSS (**b**) blends plasticized at 100 °C. Measurements were done at 100 °C as a function of applied frequency in the range of 0.5–300 rad·s^−1^, at a high applied oscillation strain of 100%, outside the linear viscoelastic region.

The Cole–Cole plots presented in Figure 3 illustrate the relationship between the storage modulus G′ and the loss modulus G″ determined over the investigated frequency range [39]. Cole–Cole analysis is widely used to evaluate the phase structure, homogeneity, and compatibility of polymer blends, providing valuable insights into the interactions between individual blend components [25]. For heterogeneous polymer systems, deviations from a single ideal semicircular curve indicate the presence of multiple relaxation processes associated with phase separation. The Cole–Cole plots support differences in viscoelastic response. Therefore, changes in the shape and continuity of the Cole–Cole curves can be used to assess the influence of additives on the morphology and viscoelastic behavior of two-phase polymer blends.

EPDM and POE are both hydrophobic polyolefin-based materials, and their blending is facilitated by their similar chemical nature. Owing to the absence of polar functional groups in their molecular structures, the two components exhibit comparable polarity, which promotes favorable interfacial interactions and enhances blend compatibility [8,9,26]. The Cole–Cole plots of all EPDM/POE systems exhibited well-defined semicircular arcs at the selected EPDM/POE ratio (Figure 3). Nevertheless, differences in the position of the arc maxima were observed among the investigated blends. These shifts may be attributed to variations in melt viscosity of the individual POE grades at 100 °C, which influence the morphology, molecular mobility, and relaxation behavior of the resulting blends, thus impacting the relationship between both moduli. The incorporation of POSS nanoparticles resulted in a noticeable shift in the arc maxima toward the position characteristic of neat EPDM. This observation suggests that POSS acts as an effective modifier of the viscoelastic response of EPDM/POE blends, reducing the differences in relaxation behavior between the blend constituents. The increased similarity of the Cole–Cole curves indicates a more homogeneous viscoelastic response in the molten state. Furthermore, due to their nanoscale dimensions and high specific surface area, POSS particles can significantly influence the rheological properties of polymer blends even at low concentrations. In particular, POSS nanoparticles, if located within the lower-viscosity POE phase, may increase the viscosity of the POE phase and enhance its elastic contribution to the overall blend response. As a consequence, an increase in the storage modulus G′ and modification of the relaxation behavior of the EPDM/POE/POSS systems can be observed. These effects highlight the dual role of POSS as both a nanoreinforcing agent and a regulator of blend morphology and viscoelastic performance.

### 3.2. Curing of EPDM/POE/POSS Blends at 160 °C

Owing to the presence of eight vinyl functional groups attached to the silsesquioxane cage, octavinyl-POSS can actively participate in peroxide-induced crosslinking reactions and may become chemically incorporated into the elastomeric network [23,25]. The vinyl groups located on the POSS molecule can undergo radical-mediated reactions, resulting in the formation of covalent bonds with polymer chains. Consequently, POSS may act not only as a nanofiller but also as a multifunctional reactive modifier capable of influencing the crosslink density, network architecture, and interfacial interactions within the blend. The high functionality of octavinyl-POSS, arising from its eight reactive vinyl groups, facilitates the formation of a more interconnected three-dimensional network, even at relatively low concentrations of dicumyl peroxide. This effect contributes to enhanced network strength and improved mechanical integrity of the cured material [40,41,42]. Our previous studies demonstrated that octavinyl-POSS participates directly in network formation and can serve as a multifunctional crosslinking node within the elastomeric structure [25]. Furthermore, octavinyl-POSS affects the curing kinetics of ethylene-propylene rubber by reducing the activation energy ($E_{a}$) of peroxide crosslinking, thereby promoting more efficient network formation and accelerating the curing process [25].

The increase in elastic torque S’ over 30 min at 160 °C was determined and the parameters of curing were calculated for EPDM/POE/POSS mixtures (Table 2).

As a reference system, an EPDM/DCP compound containing the same concentration of dicumyl peroxide (DCP) as that used in the EPDM/POE/POSS blends was prepared and evaluated. As shown in Table 2, the incorporation of POSS nanoparticles slightly reduced the incubation time t_i_ indicating an earlier onset of the curing reaction. A reduction in the optimum cure time τ_90_ was also observed. Further, higher values of the cure rate index (CRI), calculated from the maximum peaks of the first derivative of the elastic torque S’ curves (Figure S8, Supplementary Materials), were determined. The observed changes in curing resulted from the combined effects of both DCP and POSS. Synergistic contributions of peroxide-induced crosslinking and incorporation of POSS particles potentially able to participate in curing is responsible for observed changes in both τ_90_ and CRI parameters.

**Figure 4:**
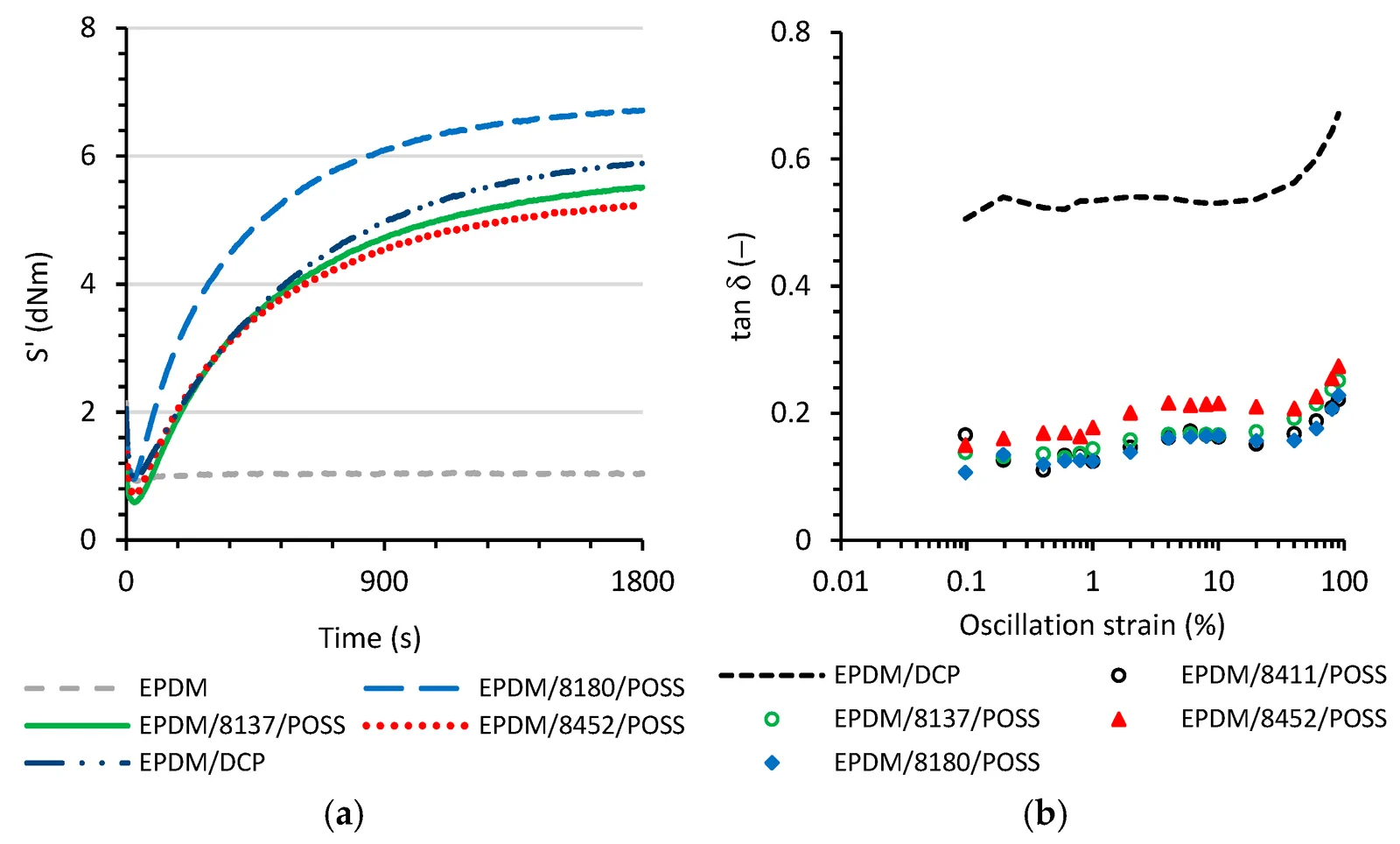
Elastic torque S’ as a function of curing time at 160 °C for EPDM, EPDM/DCP and EPDM/POE/POSS blends (**a**). Loss factor tan δ determined at 70 °C and 160 °C for EPDM/DCP and EPDM/POE/POSS samples cured for 30 min, with an applied frequency of 1 Hz (**b**).

The curing and incorporation of POSS led to an increase in the torque increment ΔS’ only for the EPDM/8180/POSS blend. In contrast, the ΔS’ values obtained for the EPDM/8137/POSS and EPDM/8452/POSS systems were comparable to those measured for the EPDM/DCP reference compound, indicating that the addition of POSS did not substantially alter the final torque development in these formulations (Table 2, Figure 4). Under identical curing conditions, the EPDM/POE blends without DCP exhibited no significant curing response, as confirmed by the elastic torque data summarized in Table S1 (Supplementary Materials). Overall, the results demonstrate that for EPDM/POE/POSS blend effect on the curing characteristics also depends on the specific POE grade incorporated into the blend (Figure 4).

Following DCP-induced crosslinking, the EPDM/POE/POSS systems can no longer be considered fully thermoplastic materials. The formation of a permanent chemical network restricts melt reprocessing ability and reduces the recyclability typically associated with conventional thermoplastic elastomers. Consequently, the cured EPDM/POE/POSS materials are more appropriately classified as partially crosslinked thermoplastic olefin-based systems (TPOs) with TPV-like characteristics rather than as conventional thermoplastic elastomers. Although the thermoplastic POE phase may still contribute to some level of processability, the presence of a crosslinked network significantly limits remelting and reprocessing compared with the uncrosslinked EPDM/POE blends.

The viscoelastic properties of the POSS-modified blends were evaluated immediately after crosslinking. Following curing at 160 °C, the samples were cooled to 70 °C, and the storage modulus G′ and loss modulus G″ were measured as a function of strain amplitude (Figure S9, Supplementary Materials). Based on these data, the damping factor tan δ was calculated and is presented in Figure 4. The test temperature of 70 °C was selected because it exceeds both the crystallization and melting temperatures of the POE phases. Under these conditions, the viscoelastic response of the material is primarily governed by the architecture of the crosslinked network and the presence of solid POSS nanoparticles rather than by crystalline contributions from the thermoplastic phase. The curing, together with the incorporation of POSS into the crosslinked EPDM/POE, enhanced the elastic response of the materials, resulting in higher storage modulus G′ values compared with EPDM cured with an equivalent amount of dicumyl peroxide. In addition, the effect on the dissipation of mechanical energy during deformation, as reflected by lower loss modulus G″ values, was observed. Consequently, the POSS-modified blends exhibited lower tan δ values than the EPDM/DCP reference system and lower values than those measured before curing (Table 1). The reduction in tan δ confirms the formation of a crosslinked structure and indicates an increased contribution of elastic deformation to the overall viscoelastic response. The more pronounced decrease in tan δ observed for the EPDM/POE/POSS blends, relative to the EPDM/DCP system, suggests the formation of a network with a distinct architecture depending on the grade of POE used. It should be noted that the primary objective of the present work was not to investigate the individual effect of DCP on the properties of the EPDM/POE blend. The reduction in tan δ is attributed to the peroxide-induced crosslinking, the incorporation of POSS nanoparticles into the crosslinked structure, and the type of POE used.

To confirm the formation of crosslinks for EPDM/POE/POSS blends, the swelling test in toluene was performed and parameters such as the equilibrium swelling Q_w_ and the percentage of sol fraction were calculated (Table S2, Supplementary Materials). All EPDM/POE samples were dissolved for 48 h in toluene. For the cured samples, the increase in sample mass due to swelling was observed. Restricted swelling and lower values of Q_w_ were observed for EPDM/POE/POSS samples compared with EPDM/DCP. A slightly lower percentage of uncured sol fraction able to be dissolved in toluene was observed for EPDM/POE/POSS blends compared with cured EPDM.

### 3.3. Dynamic Mechanical Tests at Ambient Temperature

**Figure 5:**
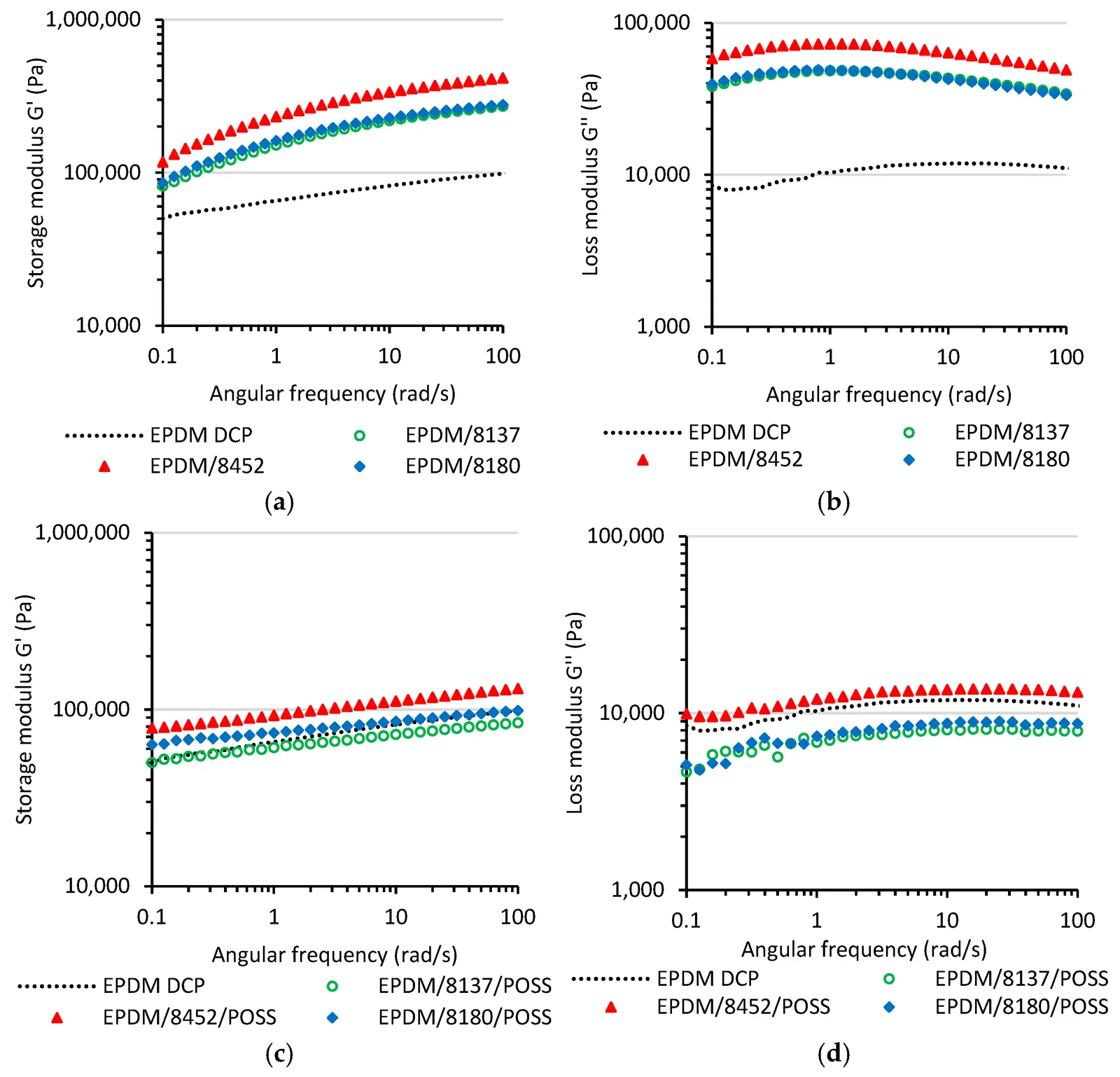
Storage shear modulus G′ and loss shear modulus G″ as a function of angular frequency for EPDM/POE blends (**a**,**b**). Storage shear modulus G′ (**c**) and loss shear modulus G″ (**d**) as a function of angular frequency for EPDM/POE/POSS (**c**,**d**) blends. Tests were done at ambient temperature, with an applied oscillation strain of 0.1%.

To gain deeper insight into the effect of the grade of POE used and POSS functionalization on the viscoelastic behavior of EPDM/POE blends, dynamic rheological measurements were performed using an ARES-G2 rotational rheometer operating in oscillatory mode. Figure 5 presents the frequency dependence of the storage modulus G′ and loss modulus G″ for the investigated EPDM/POE and EPDM/POE/POSS systems.

The presence of a crystalline phase of the thermoplastic component may have reinforced the impact on the storage shear modulus of thermoplastic/elastomer blends. POE thermoplastic elastomers are crystallizable materials. Selected POE grades differ in their crystalline phase content. Of course, incorporating POE into the elastomer phase can limit the formation of the crystalline phase compared to pure ethylene-1-octene copolymer. The ability of POE to form a crystalline phase reinforced the EPDM/POE blends; consequently, higher storage shear modulus G′ values were observed compared with DCP-cured EPDM (Figure 5a). Differences in the increase in storage modulus were also evident depending on the type of POE used. The highest G′ values were determined for the EPDM/8452 blend. This EPDM/8452 is also characterized by the highest crystalline phase content. Furthermore, the presence of the crystalline phase in the two-phase EPDM/POE material affects the ability to dissipate energy of deformation. This is confirmed by the loss modulus G″ curves as a function of frequency. The introduction of POE increases the loss modulus G″ (Figure 5b). Partial curing of EPDM/POE/POSS blends changed the viscoelastic behavior of the material (Figure 5). Crosslinking limited the crystallization capacity of the POE phase, causing a decrease in the storage modulus G′ compared with uncured EPDM/POE blends (Figure 5c). For partially crosslinked EPDM/POE/POSS systems, the reinforcement effect and enhancement of mechanical performance are governed by several factors, including the degree of crosslinking, the reinforcing action of POSS nanoparticles, and the content of the crystalline phase. Among the investigated EPDM/POE/POSS materials, the highest values of the storage modulus were observed for EPDM/8452/POSS (Figure 5c); this blend was also characterized by the highest degree of crystallinity among EPDM/POE/POSS materials. The presence of crystalline domains contributes to increased stiffness by restricting molecular mobility and acting as additional physical junction points within the polymer structure [26,43].

**Figure 6:**
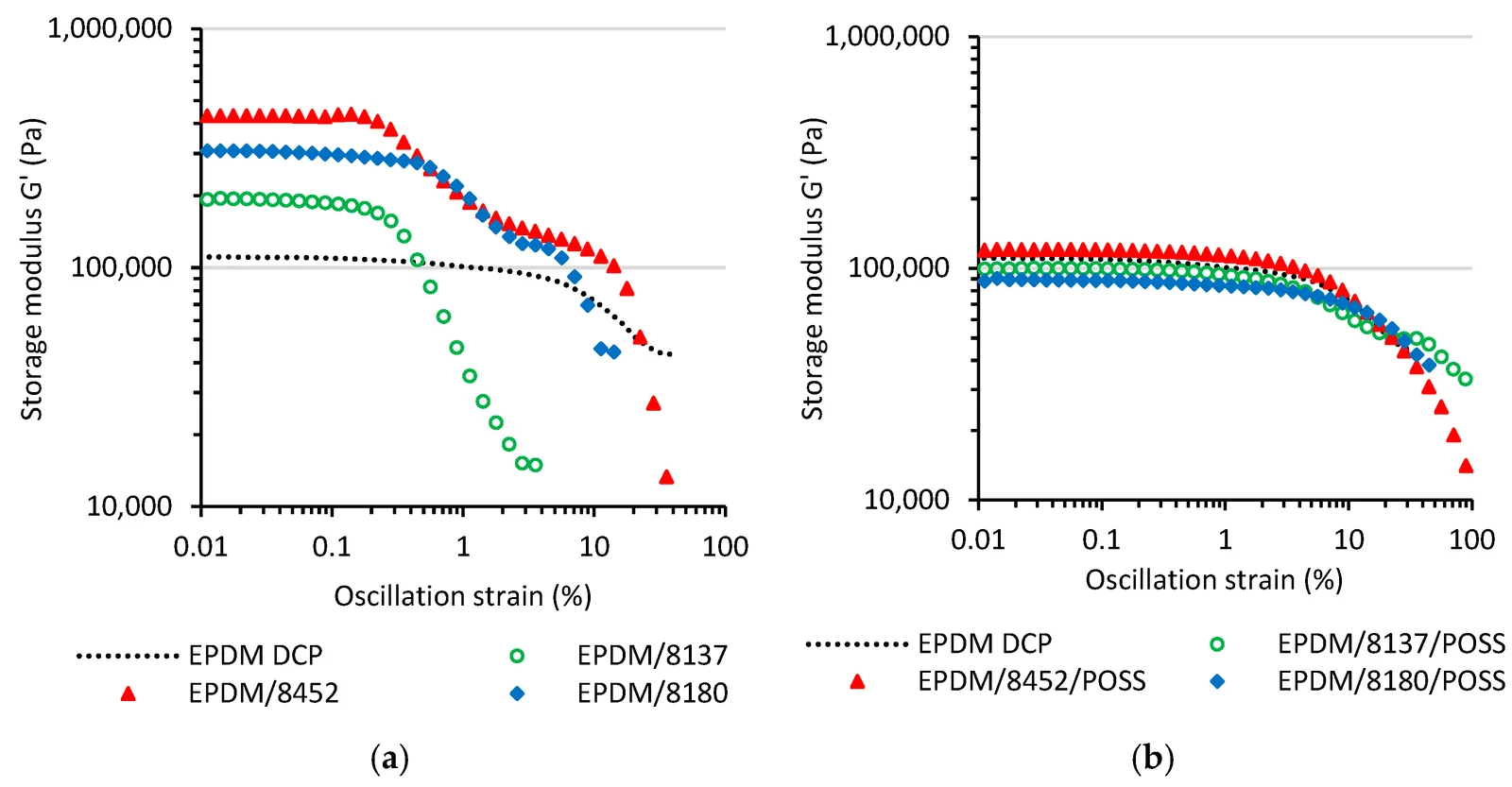
Storage shear modulus G′ as a function of oscillation strain at ambient temperature for EPDM/POE (**a**) and EPDM/POE/POSS blends (**b**). Tests were done at ambient temperature, with an applied angular frequency of 10 rad·s^−1^.

In addition, the influence of oscillatory strain amplitude on the storage modulus G′ is shown in Figure 6, providing information on the nonlinear viscoelastic response and structural stability of the blends.

One limitation of the use of Engage thermoplastic elastomers is their permanent set and inability to maintain elastic properties at high deformation values; this also applies to the storage modulus G’. This is confirmed by the curve of the measured storage modulus as a function of oscillation strain; for all EPDM/POE blends, a decrease in the G′ value started from a deformation level of approximately 1% (Figure 6a). The use of deformation higher than 1% causes chains to be pulled out of the crystalline phase, while extremely high deformation values cause chain tension and subsequent breakage. This limits the use of EPDM/POE materials as shape-memory materials. Therefore, partial crosslinking with DCP for this type of blend is justified. Partial crosslinking of the EPDM/POE/POSS blends resulted in a substantial extension of the deformation range over which the storage modulus G′ remained nearly constant (Figure 6b). This behavior is particularly advantageous for potential shape-memory applications, as it enhances the material’s ability to withstand larger deformations while maintaining adequate elastic response. Furthermore, the viscoelastic profile of the partially crosslinked EPDM/POE/POSS blends became increasingly similar to that of partially crosslinked neat EPDM, indicating that the combined effects of network formation and POSS incorporation effectively compensated for the presence of the thermoplastic phase and promoted a more elastomer-like mechanical response.

**Figure 7:**
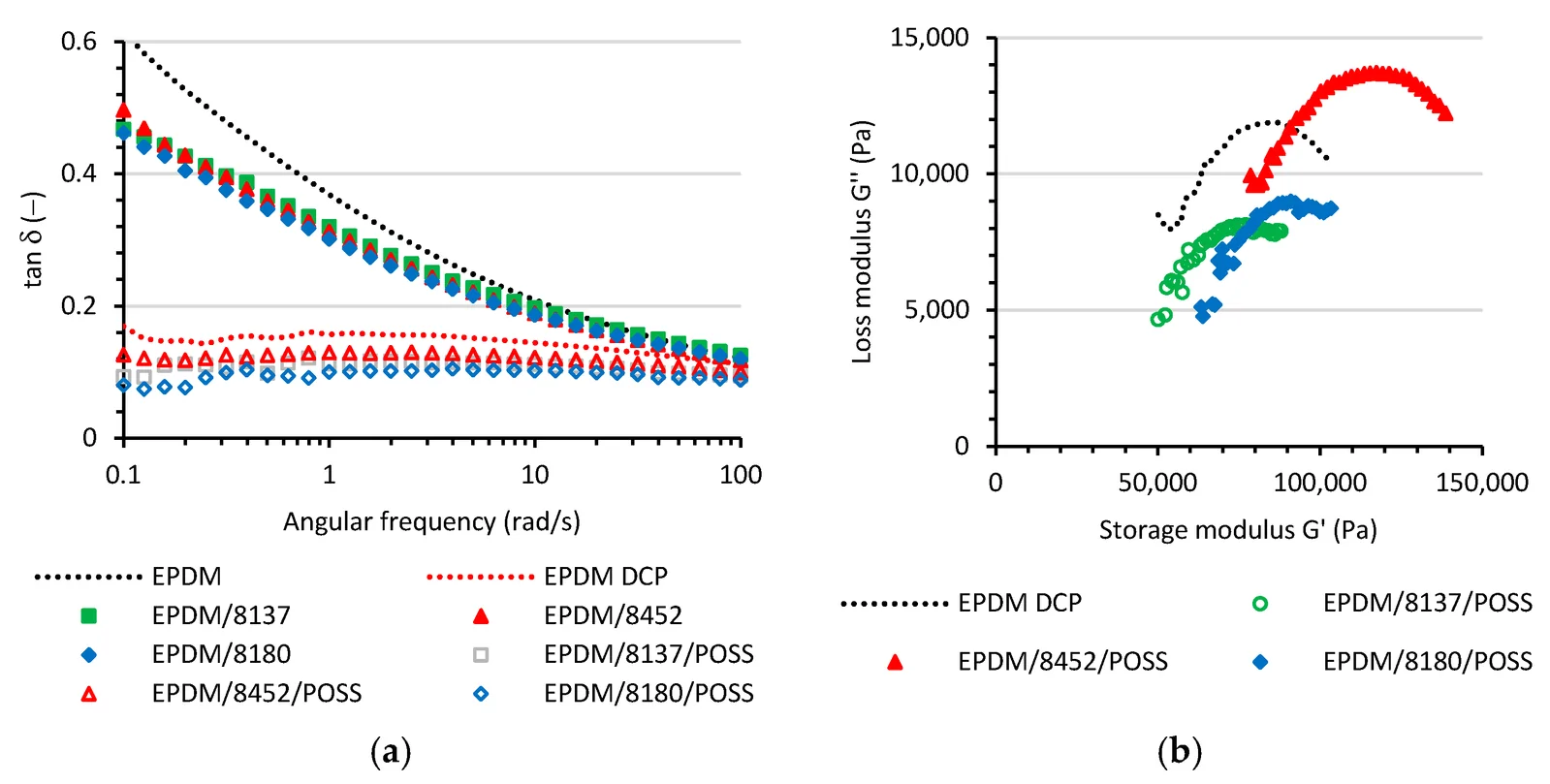
Loss factor tan δ as a function of angular frequency at ambient temperature for EPDM/POE and EPDM/POE/POSS blends, applied oscillation strain 0.1% (**a**). The Cole–Cole plots show the relationship between storage shear modulus G′ and loss shear modulus G″ for EPDM/POE/POSS blends at ambient temperature (**b**). Tests were done at ambient temperature, with a frequency range of 0.1–628 rad·s^−1^ and an applied oscillation strain of 0.1%.

Partial crosslinking of the EPDM/POE blends and the incorporation of POSS nanoparticles resulted in a reduction in the loss factor tan δ across the entire frequency range investigated (Figure 7a). The tan δ values of the EPDM/POE/POSS blends were comparable to one another and slightly lower than those observed for crosslinked EPDM.

Prior to crosslinking, both the EPDM/POE blends and neat EPDM exhibited a substantial increase in tan δ at low frequencies. This behavior is characteristic of materials with a greater ability to undergo stress relaxation over long time scales. In uncrosslinked systems, the enhanced mobility of polymer chains facilitates relaxation processes, resulting in a stronger decrease in the storage and loss moduli at low frequencies and, consequently, higher tan δ values. The incorporation of the POE phase into the EPDM matrix, however, partially restricts these relaxation mechanisms owing to the presence of crystalline domains within the thermoplastic elastomer [8,26]. These crystalline regions act as physical constraints on chain mobility, reducing stress relaxation and contributing to lower tan δ values compared with uncrosslinked neat EPDM. Following crosslinking and POSS incorporation, the combined effects of permanent network formation, nanoparticle reinforcement, and crystalline phase constraints further limit molecular mobility, resulting in a more elastic response and reduced energy dissipation throughout the investigated frequency range.

Differences in the viscoelastic behavior of the partially crosslinked EPDM/POE/POSS blends become more evident in the Cole–Cole plots (Figure 7b). The presence of well-defined semicircular arcs indicates the influence of various POE on viscoelastic response for both the EPDM/POE systems (Figure S10, Supplementary Materials) and the EPDM/POE/POSS nanocomposites (Figure 7b). Changes in the G′ and G″ relationship may be attributed to the formation of physical entanglements between EPDM and POE chains, and to the interfacial interactions and covalent linkages generated during peroxide-induced crosslinking. Variations in the position and shape of the Cole–Cole arcs observed for the EPDM/POE/POSS blends indicate differences in molecular mobility and relaxation behavior arising from changes in blend composition and network structure. These results suggest that the POE grade, curing and the incorporation of POSS influence the relaxation dynamics of the materials.

### 3.4. Static Mechanical Tests and Thermally Activated Shape-Memory Properties

To further evaluate the suitability of the developed materials for thermally activated shape-memory applications, tensile properties were investigated under static loading conditions (Table 3). The incorporation of the POE phase resulted in a slight increase in the modulus at 100% strain and tensile strength TS compared with neat EPDM rubber. This reinforcing effect may be attributed to the presence of crystalline domains within the POE phase, which contribute to load transfer and restrict molecular mobility. However, the elongation at break E_b_ of the uncured EPDM/POE blends was insufficient for efficient shape-memory operation and may limit their practical applicability in shape-programmable systems.

The substantial improvement in mechanical performance was observed for EPDM/POE/POSS blends. In particular, an approximately fourfold increase in elongation at break was observed for all EPDM/POE/POSS blends. Furthermore, the tensile strength of the EPDM/POE/POSS systems exceeded that of the peroxide-cured EPDM/DCP material. The improvements in tensile strength and elongation at break observed for EPDM/POE/POSS blends may be the result of several concurrent mechanisms, including enhanced interfacial compatibility, the reinforcing effect of POSS nanoparticle incorporation, and the increased crosslink density resulting from peroxide-induced crosslinking. However, based on the experimental data available in the present study, it is not possible to quantitatively distinguish the individual contribution of each of these factors. Peroxide crosslinking may generate network junctions within both the EPDM and POE phases as well as across the interphase region, thereby strengthening interfacial adhesion and improving stress transfer between the blend components. Simultaneously, octavinyl-POSS may contribute to the crosslinking process through its vinyl functionalities, promoting the formation of additional interchain linkages and contributing to a more homogeneous network structure. Therefore, we believe that the enhanced mechanical performance arises from the synergistic action of these mechanisms rather than from any single factor acting independently.

The primary objective of the present work was to identify the most promising EPDM/POE and EPDM/POE/DCP/POSS compositions exhibiting shape-memory behavior and to evaluate their potential as flexible shape-memory materials. Therefore, the shape-memory study should be regarded as a preliminary screening investigation aimed at selecting the most suitable formulations for further research. The shape-memory behavior of partially crosslinked EPDM/POE/POSS blends was evaluated by determining the shape fixity F and shape recovery RR ratios (Table 3). At room temperature, the dispersed POE phase contains crystalline domains embedded within the amorphous EPDM matrix. Therefore, the temperature above the melting temperature of the POE phase was selected as the transition temperature T_trans_. Samples were deformed at 80 °C. Deformation induced orientation of both EPDM and POE chains, while the partially crosslinked network restricted POE flow above its melting temperature. Following deformation, the samples were cooled under constraint to fix the temporary shape and subsequently unloaded. During cooling, partial crystallization of the POE phase occurred, stabilizing the programmed shape and limiting stress relaxation. Upon reheating above T_trans_, the POE crystallites melted, allowing the oriented chains to relax and recover their original geometry. Thus, both POE crystallinity and the crosslinked network play key roles in achieving effective thermally induced shape-memory behavior.

The values of F and RR are summarized in Table 3, while the corresponding sample lengths measured during the programming cycle are presented in Table S3 (Supplementary Materials). The results demonstrate that partial crosslinking is essential for achieving shape fixity and shape recovery performance. Uncrosslinked EPDM/POE blends exhibited significantly lower F and RR values due to irreversible deformation and flow of the POE phase during stretching. These materials also showed lower viscoelastic stability, stronger creep behavior, and a greater permanent set, all of which adversely affected shape recovery. Crosslinking in the presence of POSS improved interfacial compatibility, enhanced mechanical and viscoelastic stability, and restricted POE flow during shape programming. As a result, the EPDM/POE/POSS blends exhibited shape-memory performance despite a reduction in POE crystallinity. These findings indicate that network formation is more critical for effective shape recovery than maximizing crystalline phase content alone.

### 3.5. Thermal Properties of EPDM/POE and EPDM/POE/POSS: Glass Transition Temperature and Crystallinity of Blends

**Figure 8:**
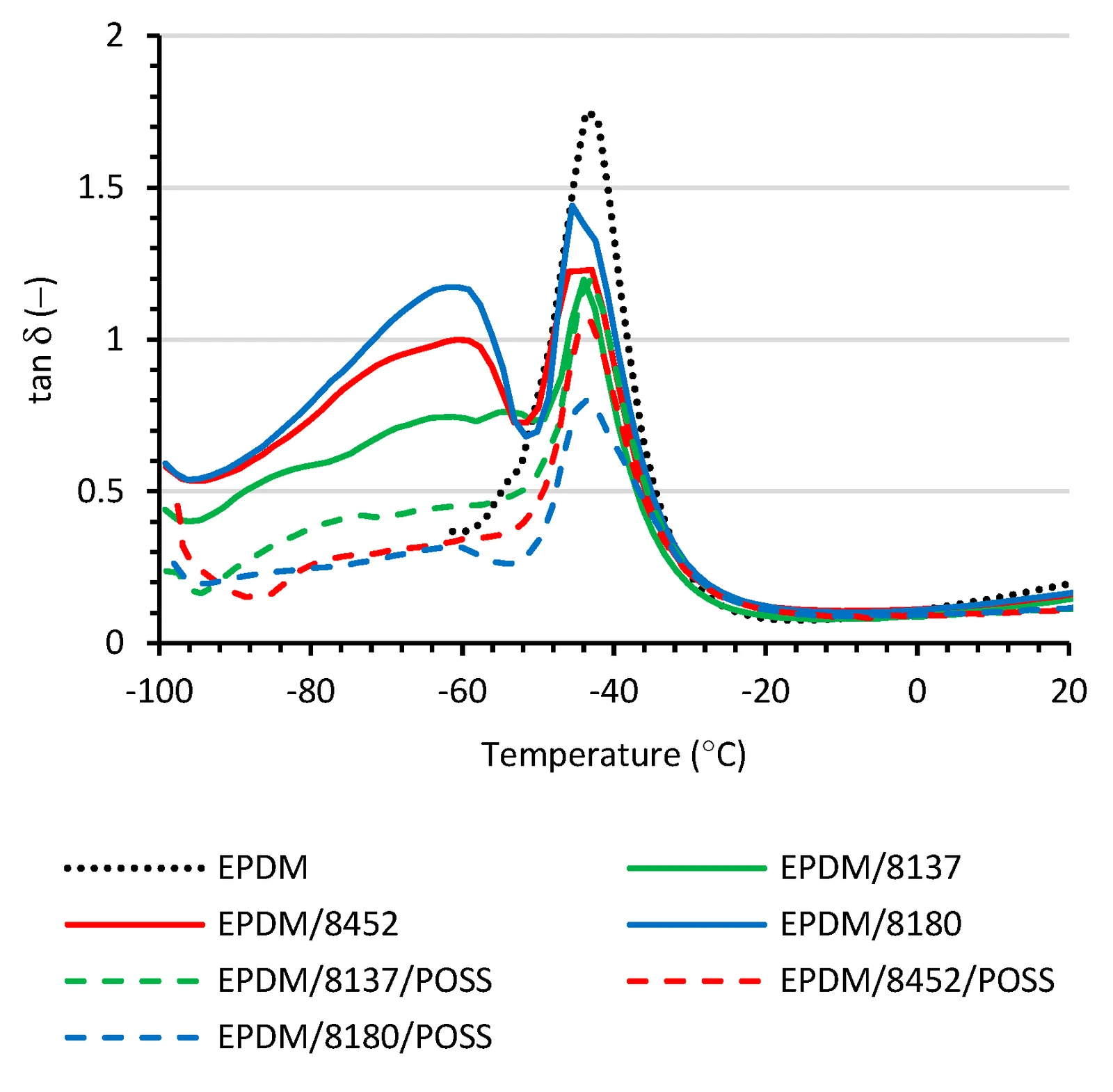
Loss factor tan δ as a function of temperature for EPDM/POE and EPDM/POE/POSS blends, with an applied oscillation strain of 0.1% and an applied frequency of 1 Hz.

The extent of compatibilization achieved through the incorporation of additives can be evaluated by analyzing changes in the glass transition temperature T_g_ of the blend components. To investigate this effect, the temperature dependence of the viscoelastic properties was measured (Figure S11, Supplementary Materials). Based on the positions of the tan δ maxima (Figure 8), the glass transition temperatures of the EPDM and POE phases were determined and are summarized in Table 4.

The EPDM/POE blends exhibited a biphasic morphology, as evidenced by the presence of two distinct glass transition temperatures T_g_ in the tan δ curves. No significant differences in the glass transition temperature of the POE phase were observed among the investigated blends, with T_g_ values consistently ranging between approximately −61 and −62 °C (Table 4, Figure 8).

The type of POE strongly influenced the height of the corresponding tan δ peak (Table 4). This effect may be attributed to differences in the degree of crystallinity of the POE grades, as an increase in crystalline phase content generally leads to a reduction in the peak intensity due to the lower fraction of amorphous material participating in the glass transition. Accordingly, Engage™ 8180 exhibited the highest tan δ peak intensity, indicating the lowest degree of crystallinity, which was subsequently confirmed by DSC measurements. The incorporation of POE had only a minor effect on the glass transition temperature of the EPDM phase, for which T_g_ remained within the range of approximately −46 to −44 °C. In contrast, partial crosslinking of the EPDM/POE blends combined with the incorporation of POSS nanoparticles resulted in a noticeable reduction in the intensity of the tan δ peak associated with the EPDM phase, accompanied by only a slight shift in the glass transition temperature. This behavior is attributed primarily to the formation of a crosslinked network, which restricts molecular mobility and reduces the amplitude of segmental motions responsible for the glass transition process [42,44]. In addition, POSS nanoparticles may further decrease the intensity of the glass transition peak through the formation of interactions or covalent bonds with polymer chains, thereby enhancing polymer-filler interactions and imposing additional constraints on chain mobility [41,42]. Similar effects have previously been reported for various elastomeric systems modified with POSS nanoparticles [21]. Our earlier studies on ethylene-propylene rubber (EPR) and hydrogenated nitrile-butadiene rubber (HNBR) functionalized with octavinyl-POSS also demonstrated a significant reduction in the intensity of the glass transition peak as a consequence of enhanced filler–polymer interactions [25].

For the EPDM/8137/POSS and EPDM/8452/POSS blends, the glass transition associated with the POE phase could not be clearly distinguished. Instead, a single broad tan δ peak with one dominant maximum was observed. This behavior suggests improved compatibility between the blend components, which may be attributed to the formation of an interphase characterized by enhanced interactions and possible covalent linkages involving EPDM, POE, and POSS. The merging of the relaxation processes into a broader transition region is consistent with increased interfacial adhesion and partial phase compatibilization induced by crosslinking and POSS incorporation. 

Differential scanning calorimetry (DSC) was employed to investigate the degree of crystallinity of the EPDM/POE blends. Two consecutive heating–cooling cycles were performed. The thermal parameters obtained during the first heating scan reflect the thermal history of the materials and the cooling conditions experienced during processing. In contrast, the second heating cycle was used to evaluate the intrinsic thermal characteristics of the blends after elimination of their previous thermal history and to assess the influence of POSS on crystallization under controlled cooling conditions. The DSC thermograms obtained from both heating–cooling cycles are presented in the Supplementary Materials (Figures S12–S15), while the thermal parameters extracted from the first heating cycle are summarized in Table S4. Table 5 compiles the melting temperature T_m_, crystallization temperature T_c_, and degree of crystallinity χ_c_. For comparison, the crystallinity values derived from the melting enthalpies obtained during the first heating cycle are also included in Table 5.

The investigated POE grades exhibited different crystallization temperatures, which were reflected in the crystallization behavior of the corresponding EPDM/POE blends. Similarly, distinct crystallization temperatures T_c_ were observed for the various EPDM/POE systems. The incorporation of POSS nanoparticles had only a minor influence on the crystallization temperature of the POE phase compared with the unmodified blends. Engage™ 8452 showed the highest T_c_ and degree of crystallinity among all systems. In contrast, the other POE grades crystallized below room temperature, indicating a lower tendency to crystallize when dispersed in the EPDM matrix. For the EPDM/8137 and EPDM/8180 blends, melting enthalpies could not be reliably determined from the second heating cycle due to weak and broad melting endotherms. Therefore, the crystallinity analysis was based on data from the first heating cycle, which more accurately reflects the morphology developed during processing and subsequent cooling under ambient conditions. POSS incorporation combined with partial crosslinking reduced the crystallinity of the POE phase and altered its melting behavior. Compared with the uncrosslinked blends, the EPDM/POE/POSS systems exhibited lower crystalline phase contents, indicating that network formation and polymer–POSS interactions restricted chain mobility and hindered crystal growth during cooling. Among the investigated materials, the EPDM/8180-based blend showed the lowest degree of crystallinity, confirming its limited crystallization capability within the EPDM matrix. Resuming the incorporation of POSS and peroxide-induced crosslinking led to a reduction in the crystallinity of the POE phase, which was accompanied by changes in viscoelastic behavior, mechanical performance, and shape-memory characteristics. These observations suggest that the crystalline domains of POE contribute to the physical network of the material and play an important role in determining its functional properties. However, because the investigated systems differ simultaneously in crystallinity, crosslink density, phase morphology, and interfacial interactions, the specific contribution of crystallinity cannot be isolated unambiguously on the basis of the current dataset.

The temperatures corresponding to 1%, 5%, and 10% mass loss, as well as the temperature associated with the maximum of the DTA peak, were determined from the TGA/DTA curves (Figure S16, Supplementary Materials) and are summarized in Table 6.

POSS nanoparticles have frequently been reported in the literature as effective additives for enhancing the thermal stability of polymeric materials [17,18]. However, such an effect was not observed for the EPDM/POE blends modified with octavinyl-POSS.

The incorporation of POSS particles had a negative impact on the thermal stability of EPDM/POE/POSS blends compared with those unmodified by POSS. Analysis of the TGA curves and the temperatures associated with 1, 5, and 10% mass loss and corresponding to the maxima of the DTA peaks (Figure S16 and Table 6) revealed that for EPDM/POE/POSS, the onset of minor mass loss occurred at lower temperatures.

The temperatures corresponding to 1 wt.% mass loss were remarkably lower for the POSS-containing systems, whereas the main thermal degradation process remained largely unaffected, as evidenced by the relatively small changes in the temperatures T_max_. The presence of POSS did not significantly affect the principal decomposition stages. Rather, it promoted an earlier onset of degradation, resulting in minor mass loss occurring at lower temperatures.

The experimental data obtained in this study do not allow the thermal degradation mechanism to be unequivocally identified. The observed differences may be associated with changes in the network structure, phase morphology, or degradation pathway induced by the presence of POSS. Detailed elucidation of these processes would require additional investigations specifically dedicated to degradation mechanisms. It should be emphasized that the primary objective of the present work was to evaluate the effect of octavinyl-POSS on the overall thermal, mechanical, rheological, and shape-memory performance of EPDM/POE blends rather than to determine the mechanism of their thermal decomposition. Furthermore, from an application perspective, an earlier onset of minor mass loss in the vicinity of ~240 °C does not preclude the use of these materials in the proposed applications, since their intended service temperatures are substantially lower than the temperatures at which thermal degradation occurs. Therefore, the observed reduction in T_1%_ is not expected to adversely affect the practical performance of the investigated materials.

### 3.6. Relaxation and Creep Recovery of EPDM/POE and EPDM/POE/POSS Blends

Stress relaxation is a characteristic feature of elastomeric and viscoelastic polymer systems, reflecting their ability to dissipate stresses generated during deformation. Relaxation is a complex, multiscale process involving several concurrent mechanisms that operate over different time scales [25]. These mechanisms may include molecular chain rearrangement and reorientation, segmental motions of polymer chains, disruption of crystalline or phase-separated microdomains, weakening and rupture of interfacial interactions, and the relaxation of network constraints in crosslinked systems.

In multiphase polymer blends, the stress relaxation behavior is strongly governed by the material’s microstructure, including phase morphology, interfacial characteristics, and the strength of interactions between the constituent phases. A higher degree of phase compatibility and a more homogeneous morphology generally result in stronger interfacial adhesion, which restricts molecular mobility and alters the mechanisms of energy dissipation. Consequently, the nature of the interphase plays a crucial role in determining the relaxation dynamics and overall viscoelastic response of the material.

**Figure 9:**
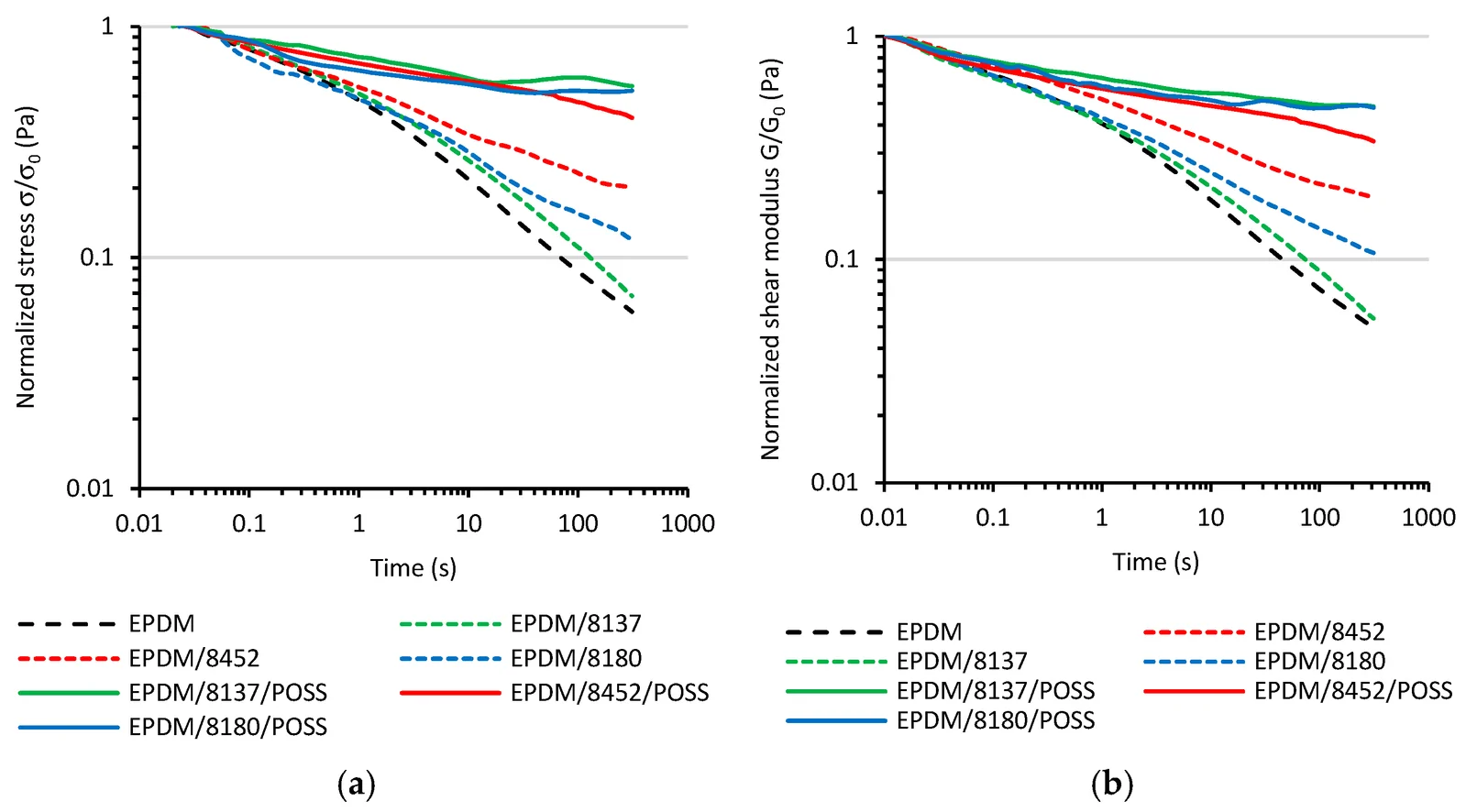
The relaxation curves normalized with respect to the initial stress σ_0_ and initial modulus G_0_ plots for EPDM/POE (**a**) and EPDM/POE/POSS blends (**b**).

The plots for stress σ and shear modulus G decay measured as a function of time are shown in Figures S17 and S18 (Supplementary Materials). The plots show typical relaxation behavior, with fast stress or modulus G decay at the beginning of the process, which slows down with time. The highest initial stress σ_o_ and modulus G_o_ were observed for neat EPDM. The presence of the secondary POE phase reduced the generated stress under applied strain. Furthermore, the grade of the POE used influenced the value of initial stress σ_o_ and modulus G_o_. The morphology of EPDM/POE blends differs due to various grades of ethylene-1-octene copolymer used. The type of Engage used can affect the size of the formed droplets but also has an impact on the interphase. Among EPDM/POE blends, the highest initial stress σ_o_ was determined for the EPDM/8137 blend. Although the highest energy was stored in deformed chains for this blend, the fastest stress decay was observed, as shown in the normalized relaxation curves (Figure 9).

The initial decrease in stress is primarily associated with rapid relaxation mechanisms, such as the reorientation of polymer chain segments and localized motions of phase domains present in both components of the blend [45,46]. At longer relaxation times, slower relaxation processes become dominant and may arise from long-range molecular chain rearrangements, particularly within the interfacial region between the EPDM and POE phases. These processes may additionally involve the gradual weakening of physical interactions at the phase boundary, contributing to the continued decay of stress [8,46]. Thus, factors that can disturb the relaxation include the morphology of the blend, the size of the POE droplets, and the crystallinity, which limits the mobility of polymeric chains [26,43,45]. The stress generated under strain relaxation can be dissipated or stored in deformed chains if they are not able to retract due to the presence of crosslinks [42,44]. The incorporation of POSS in the blend network, together with partial curing, has a meaningful influence on the degree of relaxation of EPDM/POE blends, as it is visible in the normalized plots with respect to the initial stress or modulus plots (Figure 9). During the initial stage of relaxation (up to approximately 1 s), the stress decay behavior was similar for both the uncured EPDM/POE blends and the corresponding POSS-modified and cured systems. However, pronounced differences between cured and uncured materials became apparent at longer relaxation times. In particular, the EPDM/POE/POSS blends exhibited a slower decrease in relaxation stress, indicating enhanced resistance to stress relaxation. As expected, the combined effect of peroxide-induced crosslinking and POSS incorporation resulted in a reduced relaxation rate and lower stress decay over the same time interval. This behavior may be attributed to the formation of a three-dimensional network and the presence of POSS particles, both of which significantly restricted the mobility of the polymer chains. The influence of curing on the relaxation behavior was observed for all EPDM/POE/POSS systems; however, the effect was particularly pronounced for the EPDM/8137/POSS and EPDM/8180/POSS blends. For these materials, relaxation equilibrium was reached within the measurement period, and nearly constant normalized stress values were observed after approximately 100 s. In contrast, the EPDM/8452/POSS blend exhibited a continuous decrease in stress throughout the entire test duration, resembling the relaxation behavior of the uncured EPDM/POE blends.

**Figure 10:**
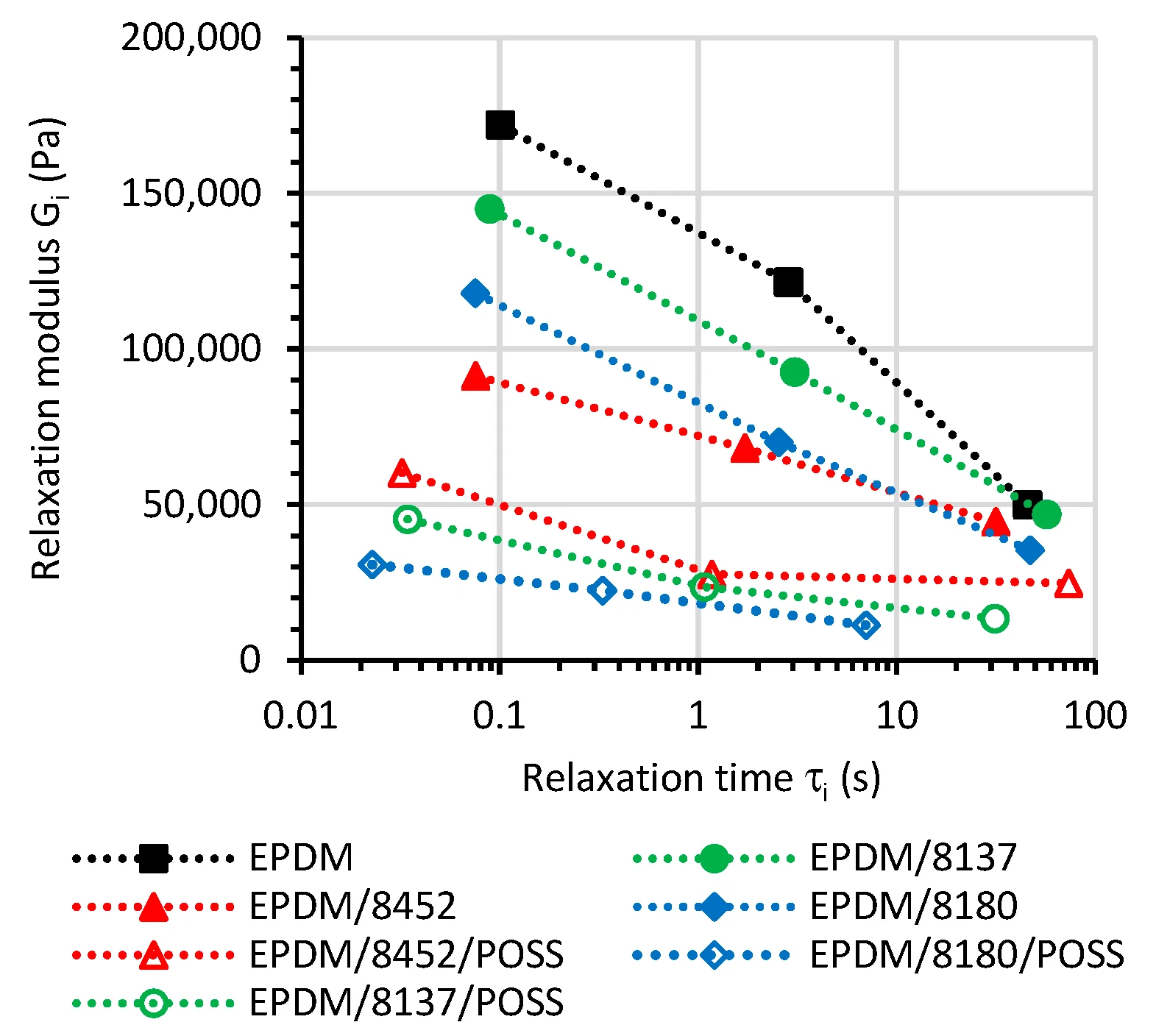
The relaxation modulus G_i_ and relaxation times τ_i_ calculated based on the Maxwell equations (Equation (3)).

The modulus values G(t) obtained from the stress relaxation measurements were fitted using the generalized Maxwell model. The resulting pairs of fitted parameters, namely the relaxation moduli G_i_ and relaxation times τ_i_, are presented in Figure 10. The numerical values of the fitted Maxwell parameters, calculated according to Equation (3), are summarized in Table S5 (Supplementary Materials). Figure 10 illustrates the relationship between the relaxation moduli and their corresponding relaxation times for the investigated materials. The fitted G_i_ − τ_i_ pairs reveal distinct differences in the relaxation behavior of materials with different compositions. The short-term relaxation processes, represented by τ_1_ and τ_2_, are generally associated with local molecular motions, such as the rotation of pendant groups and the movement of short polymer chain segments. Higher values of τ_1_ and τ_2_ were observed for the unmodified EPDM/POE blends. This behavior can be attributed to their higher degree of crystallinity, which restricts local chain mobility due to the incorporation of chain segments into crystalline domains, thereby prolonging the characteristic relaxation times as compared with neat EPDM. The long-term relaxation process, represented by τ_3_, is associated with the mobility of larger polymer chain segments, including chain portions located between crosslinking points, and the disruption and subsequent reorganization of intermolecular and interfacial interactions within the composite structure. The presence of POSS nanoparticles additionally affects the relaxation dynamics by influencing the transition between successive relaxation mechanisms. This effect is reflected in the altered distribution of the G_i_ − τ_i_ pairs and the flatter profile of the relaxation spectrum observed for the modified composites, indicating a broader distribution of relaxation processes. Crosslinking exerted a particularly pronounced influence on the equilibrium modulus G_e_, the values of which are presented in Table S5 (Supplementary Materials). Lower G_e_ values were determined for the uncured EPDM/POE blends. The formation of a crosslinked network may increase material stiffness and limit long-range molecular mobility, thereby reducing the extent of stress relaxation and resulting in higher equilibrium modulus values. In contrast, although the uncured blends contain a greater fraction of crystalline domains that partially restrict chain motion, the absence of permanent network junctions allows more extensive molecular rearrangement during relaxation. Consequently, these materials exhibit lower G_e_ values than their crosslinked counterparts. Overall, the Maxwell model analysis confirms that both crystallinity and crosslink density govern the relaxation behavior of EPDM/POE-based systems. While crystalline regions primarily affect short-term relaxation processes by limiting local molecular motions, the crosslinked network predominantly controls long-term relaxation behavior and determines the magnitude of the equilibrium modulus. The incorporation of POSS nanoparticles further modifies the relaxation spectrum by introducing additional constraints on polymer chain mobility and altering the microstructural organization of the composites.

Dimensional stability is a key requirement for thermally responsive shape-memory polymers. Creep-recovery testing is widely used to evaluate dimensional stability by characterizing the viscoelastic response of a material under constant stress [47,48,49]. During the creep stage, the material undergoes an instantaneous elastic deformation followed by time-dependent viscoelastic and viscous deformation. Upon stress removal, rapid elastic recovery is accompanied by a slower viscoelastic recovery process. The residual strain remaining after recovery, referred to as unrecovered creep or permanent set, represents irreversible deformation and provides a measure of the material’s dimensional stability and shape-retention capability.

**Figure 11:**
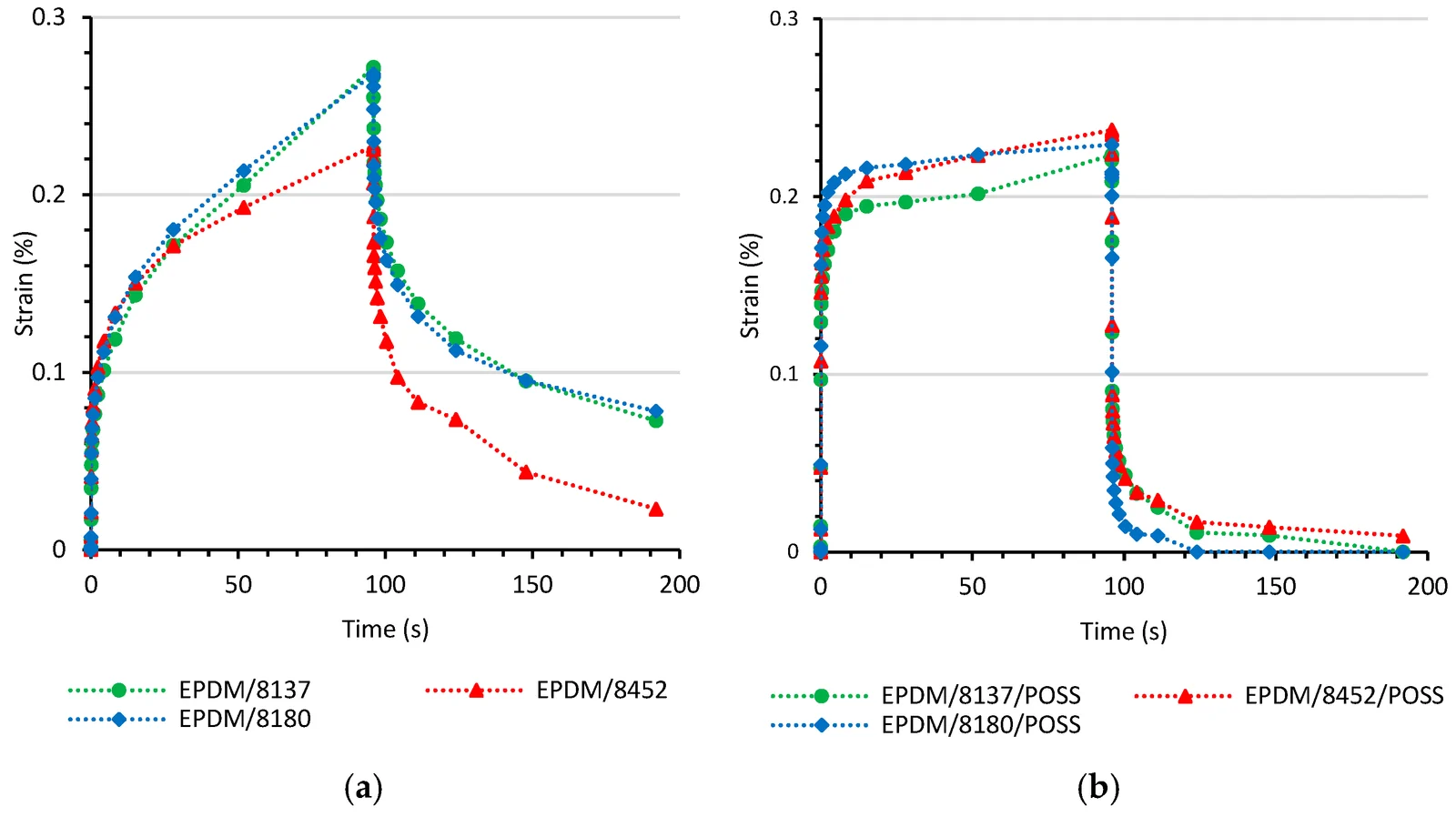
Creep and recovery plots for EPDM/POE (**a**) and EPDM/POE/POSS (**b**).

The creep-recovery behavior of the EPDM/POE and EPDM/POE/POSS blends is presented in Figure 11, while the corresponding curve for neat EPDM rubber is shown in Figure S19 (Supplementary Materials). The plots of creep compliance, recovery compliance and recovery strain are shown in Figures S20–S22 (Supplementary Materials); the calculated values of recovery percentages are compiled in Table S6 (Supplementary Materials). For all EPDM/POE blends, the creep stage begins with an instantaneous increase in strain resulting from the elastic response of the material to the applied stress. This initial deformation is followed by a time-dependent viscoelastic response associated with molecular chain mobility and structural rearrangements within the polymer matrix. As the loading period progresses, viscous flow becomes increasingly pronounced, leading to a gradual increase in strain. Among the investigated systems, the EPDM/8452 blend exhibited the lowest creep deformation and the smallest contribution of viscous flow. Moreover, for this blend, the lowest values of creep compliance J and the highest values of recoverable compliance J_rec_ are determined (Figures S20 and S21, Supplementary Materials). This behavior is likely related to its higher degree of crystallinity, which restricts molecular mobility and limits chain rearrangement under an applied load. Enhanced dimensional stability and shape recovery of the EPDM/8452 blend are further confirmed by the higher recovery strain value ε_rec_—representing the portion of deformation that disappears upon load removal (Figure S22, Supplementary Materials)—and the higher calculated recovery percentage (Table S6, Supplementary Materials). The incorporation of POSS nanoparticles combined with peroxide-induced crosslinking remarkably altered the creep behavior of the blends. Compared with the corresponding unmodified EPDM/POE systems, the EPDM/POE/POSS nanocomposites exhibited a substantially lower creep strain. The formation of a partially crosslinked network, together with the presence of POSS particles, effectively restricted chain mobility and suppressed viscous flow under the applied stress. Furthermore, the lower creep compliance J, together with higher recovery compliance J_rec_, was observed for EPDM/POE/POSS blends (Figures S20 and S21, Supplementary Materials). During the recovery stage, the materials undergo time-dependent molecular relaxation as they attempt to regain their original dimensions. This behavior is particularly important in the context of shape-memory applications, where the recovery of the programmed shape is a key performance criterion. Excessive viscous deformation and the resulting permanent set contribute directly to a reduction in the shape recovery ratio (RR). Consequently, the EPDM/POE blends, which exhibited significant viscous flow during the creep stage, failed to achieve complete strain recovery within the experimental time window. In contrast, the EPDM/POE/POSS nanocomposites demonstrated nearly complete strain recovery over the same recovery period, indicating substantially improved dimensional stability. This was also confirmed by higher values of recovery percentage R, 96.2% for EPDM/8452/POSS, 97.8% for EPDM/8137/POSS, and 99.8% for EPDM/8180/POSS, compared with uncured EPDM/POE blends. The reduced permanent set observed for these materials suggests that the crosslinked network and nanoparticle reinforcement effectively limit irreversible deformation. From an engineering perspective, the permanent deformation observed in the unmodified EPDM/POE blends may compromise long-term dimensional stability under sustained loading conditions. The incorporation of POSS and the development of a partially crosslinked structure significantly enhance creep resistance, promote faster recovery, and minimize residual deformation, thereby improving the suitability of these materials for shape-memory and other load-bearing applications.

### 3.7. Morphology of EPDM/POE/POSS Blends Before and After Crosslinking

**Figure 12:**
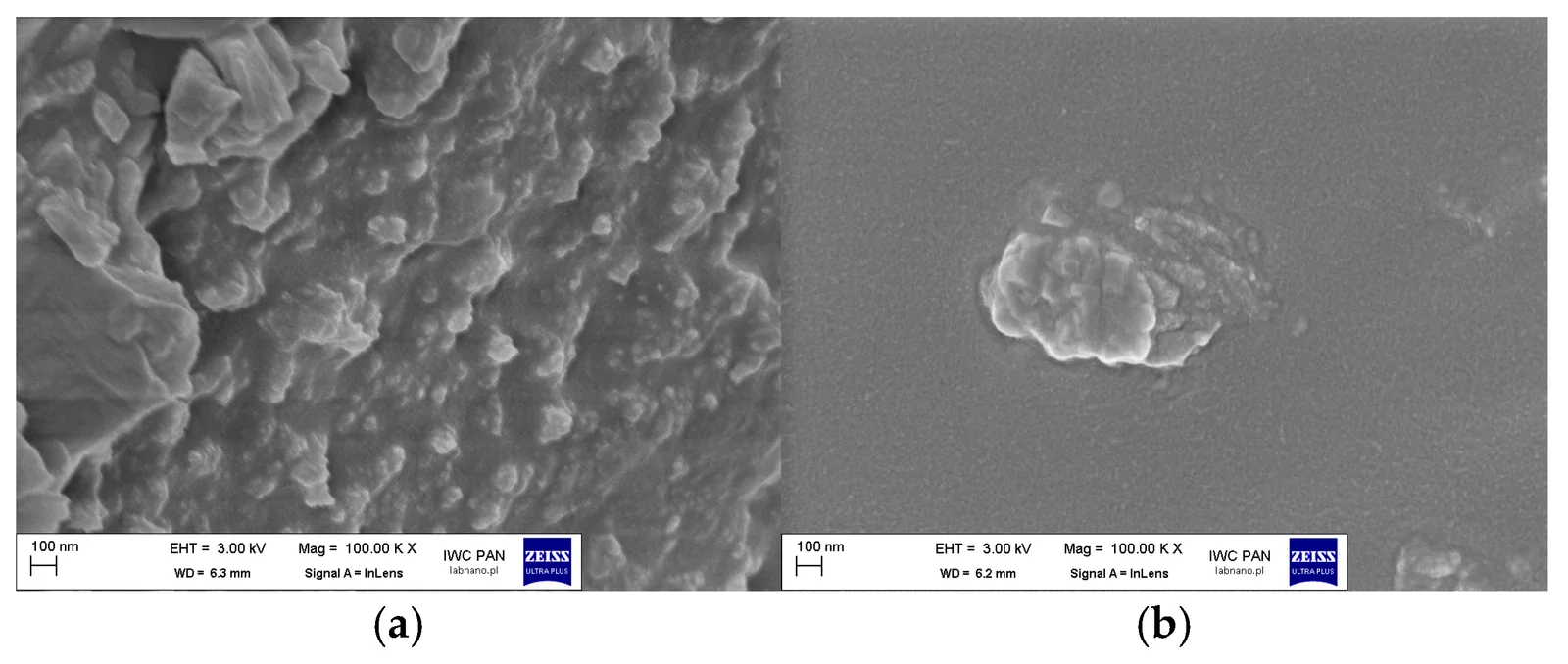
SEM pictures of EPDM/8137/POSS blends before (**a**) and after curing (**b**).

**Figure 13:**
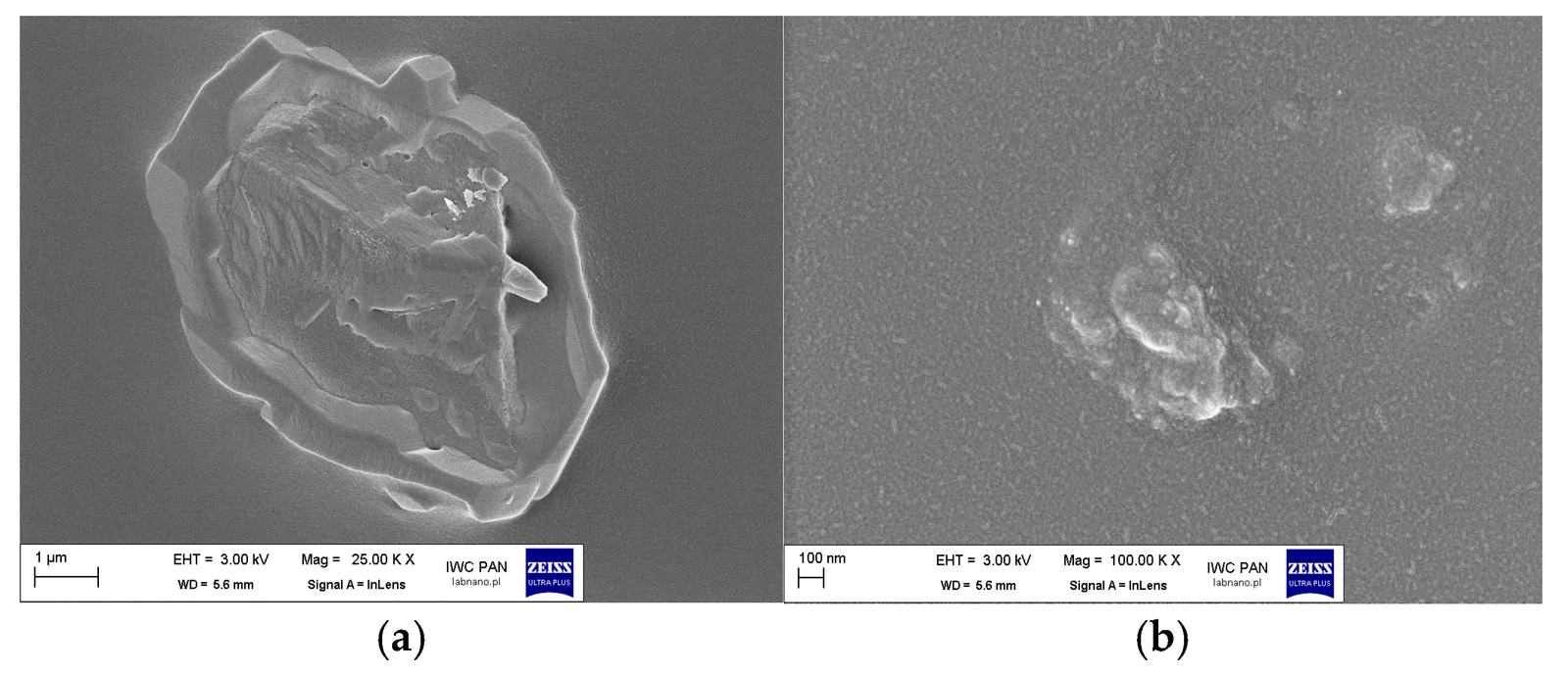
SEM pictures of EPDM/8452/POSS blends before (**a**) and after curing (**b**).

**Figure 14:**
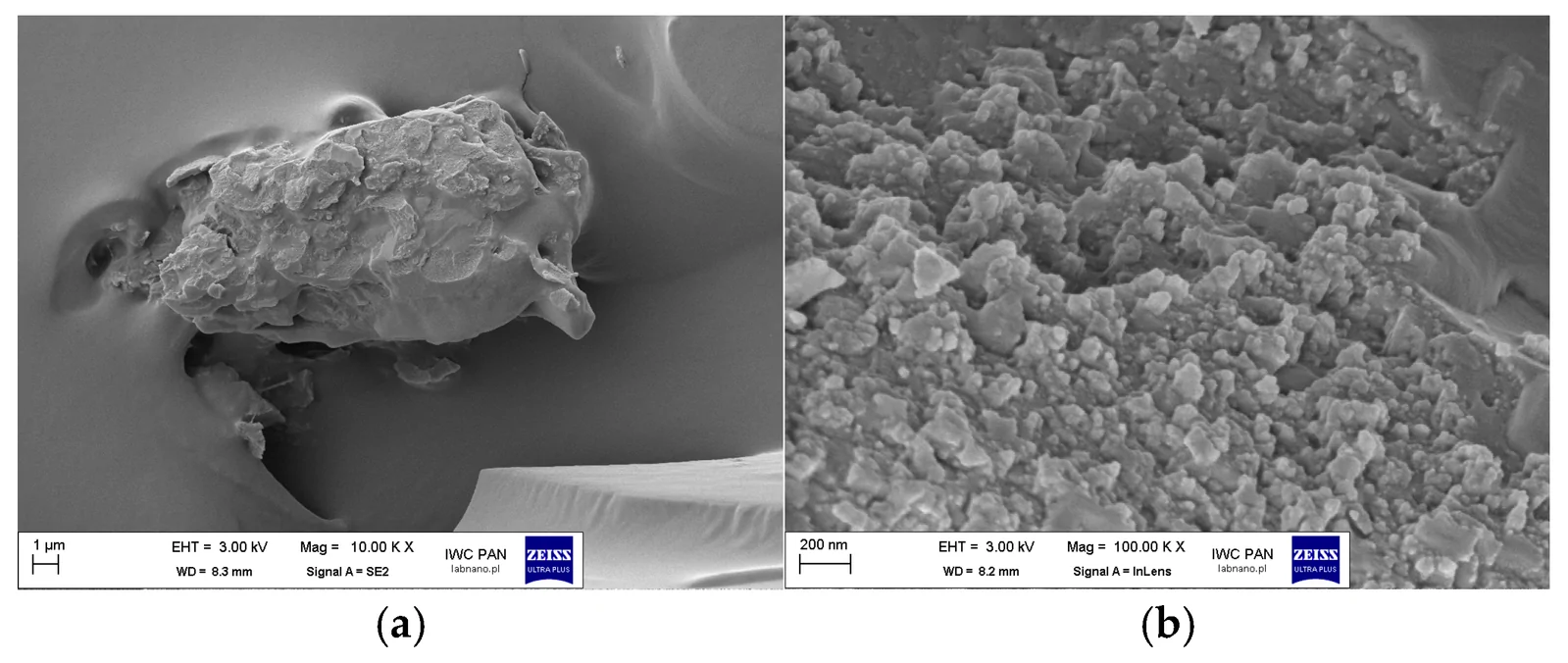
SEM pictures of EPDM/8180/POSS blends before (**a**) and after curing (**b**).

Scanning electron microscopy (SEM) was employed to investigate the morphology of the EPDM/POE/POSS blends before and after crosslinking. The micrographs revealed a distinct two-phase morphology in the uncrosslinked systems, characterized by the presence of dispersed POE domains within the EPDM matrix (Figure 12, Figure 13 and Figure 14). For the EPDM/8137/POSS blend, domains of the Engage 8137 thermoplastic elastomer were clearly visible prior to crosslinking (Figure 12). Following the crosslinking process, the morphology became noticeably more homogeneous, indicating improved interfacial compatibility between the blend components, although individual POE domains could still be identified. As shown in Figure 12, the morphology of the EPDM/8137/POSS blend changed after curing. This observation suggests that curing, together with the incorporation of POSS, contributed to interfacial compatibilization and participated in the crosslinking process, thereby promoting stronger interactions between the blend components. The improved compatibility of the EPDM/8137/POSS system is further supported by our other results, which revealed the presence of a single broad glass transition temperature T_g_, indicative of increased interphase interactions.

The morphology of the blends was strongly influenced by the characteristics of the POE grade employed, particularly its melt viscosity and crystallization behavior. In the EPDM/8452/POSS blend, larger POE domains were observed prior to crosslinking, accompanied by a clearly defined phase boundary, indicating limited compatibility between the EPDM matrix and the thermoplastic elastomer phase (Figure 13a). Following crosslinking in the presence of POSS, the interfacial region became significantly more homogeneous, suggesting enhanced interfacial adhesion and improved compatibilization of the blend. Nevertheless, isolated POSS agglomerates were still observed within the matrix, as illustrated in Figure 13.

In contrast, the EPDM/8180/POSS blend retained a pronounced two-phase morphology even after crosslinking (Figure 14). This observation is consistent with the thermal analysis results, for which two distinct glass transition temperatures were detected, confirming the persistence of phase separation within the material. The observed reduction in domain size and improvement in interfacial homogeneity result from crosslinking and POSS incorporation. Differences in phase morphology and interfacial interactions are likely responsible for the variations observed in the Cole–Cole plots after crosslinking. Furthermore, the blend morphology plays a key role in determining molecular mobility and relaxation behavior, thereby influencing the overall rheological response of the EPDM/POE/POSS systems.

## 4. Conclusions

The incorporation of ethylene-1-octene copolymers (POEs) into the EPDM matrix enabled effective tailoring of the rheological, viscoelastic, and mechanical properties of the resulting blends.

The melt viscosity and crystallinity of the POE phase were identified as the key factors governing blend morphology and performance.

Regardless of POE grade, the addition of POE reduced the complex viscosity of EPDM at 100 °C, promoted shear-thinning behavior, and decreased the storage modulus of the melt. The incorporation of octavinyl-POSS modified the rheological response of melted blends, reducing viscosity at low shear rates while maintaining favorable flow behavior before curing.

At ambient temperature, the type of POE used affects the viscoelastic response of EPDM/POE and the relationship between storage and loss modulus, as confirmed by changes in the shape of Cole–Cole plots. Partial crosslinking of the EPDM/POE/POSS systems changed their mechanical and viscoelastic performance at ambient temperature. Although crosslinking reduced the crystallinity of the POE phase and consequently lowered the storage modulus, it significantly increased the strain range over which elastic behavior was maintained, while enhancing tensile strength TS and elongation at break E_b_. In addition, the crosslinked blends exhibited slower stress relaxation, reduced creep, and improved dimensional stability. The influence of peroxide-induced curing together with the incorporation of POSS on glass transition temperatures, particularly for the EPDM/8137/POSS and EPDM/8452/POSS systems, was confirmed by the appearance of a single broad glass transition peak.

Despite their lower crystallinity, the partially cured EPDM/POE/POSS blends exhibited higher shape fixity F and shape recovery RR ratios than the corresponding uncrosslinked EPDM/POE systems.

Looking forward, future research should focus on further optimization of the EPDM/POE/POSS system through the evaluation of alternative crosslinking systems, for example, by exploring eco-friendly crosslinkers or co-agents to substitute for or minimize the reliance on dicumyl peroxide. Additionally, scaling up the mixing and vulcanization processes using continuous industrial extrusion techniques will be an essential step toward using these high-performance shape-memory materials in commercial applications.

## Figures and Tables

**Table 1:** Viscoelastic parameters at processing temperature of 100 °C: storage shear modulus G’, loss shear modulus G’’, loss factor tan δ, complex viscosity η*, dynamic viscosity η’.

	G’ kPa (▲)	G’’ kPa (▲)	Tan δ − (▲)	η* kPa·s (▲)	η’ kPa·s (▲)
EPDM	100.4 ± 1.9	80.2 ± 0.9	0.80 ± 0.01	40.9 ± 0.6	25.5 ± 0.3
EPDM/8137	79.0 ± 1.0	78.3 ± 0.5	0.99 ± 0.02	35.4 ± 0.2	24.9 ± 0.2
EPDM/8137/POSS	68.5 ± 0.8	71.3 ± 0.6	1.04 ± 0.01	31.5 ± 0.2	22.7 ± 0.2
EPDM/8452	86.0 ± 0.5	79.2 ± 0.5	0.92 ± 0.01	37.2 ± 0.2	25.2 ± 0.2
EPDM/8452/POSS	70.5 ± 1.5	71.2 ± 1.0	1.01 ± 0.01	31.9 ± 0.5	22.7 ± 0.3
EPDM/8180	115.0 ± 0.9	98.0 ± 0.6	0.85 ± 0.01	48.1 ± 0.3	31.2 ± 0.2
EPDM/8180/POSS	89.6 ± 1.5	79.3 ± 1.4	0.89 ± 0.01	38.1 ± 0.6	25.2 ± 0.4

(▲) mean values determined after plasticization of rubbery mixtures at 100 °C. Measurements were performed using 0.5 Hz (3.14 rad·s^−1^) and 0.2% oscillation strain, equivalent to low shear rates (0.08 s^−1^).

**Table 2:** Parameters of curing: minimum elastic torque S’_min_, maximum elastic torque S’_max_, increase in elastic torque during curing ΔS’, incubation time t_i_, time of 5% elastic torque increase TC_5_, optimal curing time t_90_, and cure rate index CRI.

	S’_min_ dNm	S’_max_ dNm	ΔS’ dNm	t_i_ s	TC_5_ min	t_90_ min	CRI dNm·min^−1^
EPDM	0.92	1.04	0.11	-	-	-	-
EPDM/DCP	0.93	5.90	4.97	36	1.16	20	0.58
EPDM/8137/POSS	0.59	5.51	4.93	32	1.17	18	0.73
EPDM/8452/POSS	0.70	5.24	4.54	32	1.02	18	0.75
EPDM/8180/POSS	0.95	6.72	5.76	29	0.84	16	1.15

The increment of elastic torque was calculated according to ΔS’ = S’_max_ − S’_min_. The measurements were done at 160 °C, with an applied frequency of 1.67 Hz and an oscillation strain of 7%.

**Table 3:** Mechanical and shape-memory properties, modulus at 100% deformation SE_100_, tensile strength TS, elongation at break E_b_, shape fixity F, and shape recovery RR, determined for EPDM/POE and EPDM/POE/POSS blends.

	SE_100_ MPa	TS MPa	E_b_ %	F %	RR %
EPDM	-	0.35 ± 0.02	62 ± 15	-	-
EPDM/8137	0.60 ± 0.01	0.60 ± 0.01	101 ± 5	66.7 ± 6.2	32.0 ± 6.1
EPDM/8452	0.78 ± 0.01	0.79 ± 0.01	115 ± 5	64.0 ± 3.6	29.1 ± 6.1
EPDM/8180	0.63 ± 0.02	0.67 ± 0.03	126 ± 1	64.3 ± 1.8	30.6 ± 3.6
EPDM/DCP	0.51 ± 0.07	0.95 ± 0.25	373 ± 64	-	-
EPDM/8137/POSS	1.14 ± 0.04	2.36 ± 0.17	453 ± 38	89.8 ± 3.6	93.9 ± 2.5
EPDM/8452/POSS	1.53 ± 0.06	2.89 ± 0.09	459 ± 26	94.2 ± 3.6	96.1 ± 3.0
EPDM/8180/POSS	1.19 ± 0.02	2.68 ± 0.01	437 ± 20	95.5 ± 2.2	96.2 ± 2.6

**Table 4:** The glass transition temperatures for EPDM and POE phases, and the values of tan δ at maximum glass transition peaks.

	T_g_^EPDM^ °C	Tan δ−	T_g_^POE^ °C	Tan δ−
EPDM	−43.7	1.75	---	---
EPDM/8137	−44.0	1.20	−61.2	0.75
EPDM/8452	−44.5	1.23	−60.7	0.99
EPDM/8180	−45.5	1.44	−62.1	1.17
EPDM/8137/POSS	−42.9	1.20	---	---
EPDM/8452/POSS	−43.0	1.06	---	---
EPDM/8180/POSS	−43.6	0.80	−60.1	0.32

**Table 5:** Parameters determined based on DSC tests: temperature of crystallization T_c_, enthalpy of crystallization ΔHc, melting temperature T_m_, enthalpy of melting −ΔH_m_, degree of crystallinity calculated based on II cycle of heating χ_c_, melting temperature determined based on I cycle of heating T_m_^I cycle^, degree of crystallinity calculated based on I cycle of heating χ_c_^I cycle^.

Sample	T_m_^I cycle^ °C (▲)	χ_c_^I cycle^ % (▲)	T_c_ °C	ΔH_c_ mJ × g^−1^	T_m_ °C	−ΔH_m_ mJ × g^−1^	χ_c_ %
EPDM	51.0	1.46	13.60	2.61	-	-	-
EPDM/8137	52.2	9.29	17.02	4.11	-	-	-
EPDM/8452	74.2	14.43	33.04	7.94	71.59	5.88	10.14
EPDM/8180	49.8	8.19	10.81	2.10	-	-	-
EPDM/8137/POSS	42.4	5.02	18.78	4.31	54.49	2.80	4.83
EPDM/8452/POSS	71.2	5.78	33.48	7.80	68.50	5.03	8.67
EPDM/8180/POSS	42.7	2.36	10.25	3.06	48.38	1.88	3.24

(▲) values were calculated based on the first cycle of sample heating.

**Table 6:** Temperatures corresponding to 1, 5, and 10% weight loss T_1, 5, 10%_, and temperature at maximum DTA peak T_max_.

	T_1%_ °C	T_5%_ °C	T_10%_ °C	T_max_ °C
EPDM	328	445	454	483
EPDM/8137	403	445	454	483
EPDM/8452	412	445	454	488
EPDM/8180	412	445	454	487
EPDM/8137/POSS	249	436	450	483
EPDM/8452/POSS	240	431	445	488
EPDM/8180/POSS	375	431	445	483

## Data Availability

The original contributions presented in this study are included in the article/Supplementary Material. Further inquiries can be directed to the corresponding author.

## Supplementary Materials

The following supporting information can be downloaded at https://www.mdpi.com/article/10.3390/ma19194216/s1. Scheme S1: The procedure applied during a single shape-memory test. Figure S1: Complex viscosity η* (Pa·s) at 100 °C for plasticized EPDM/POE and EPDM/POE/POSS blends. Figure S2: Storage modulus G′ (kPa) at 100 °C for plasticized EPDM/POE and EPDM/POE/POSS blends. Figure S3: Loss modulus G″ (kPa) at 100 °C for plasticized EPDM/POE and EPDM/POE/POSS blends. Figure S4: Loss factor tan δ (−) at 100 °C for plasticized EPDM/POE and EPDM/POE/POSS blends. Figure S5: Complex viscosity η* as a function of applied frequency (Hz) at processing temperature 100 °C for EPDM/POE and EPDM/POE/POSS blends, applied high oscillation strain 100%. Figure S6: Storage modulus G′ (kPa) as a function of applied frequency (Hz) at processing temperature 100 °C for EPDM/POE and EPDM/POE/POSS blends, applied high oscillation strain 100%. Figure S7: Loss modulus G″ (kPa) as a function of applied frequency (Hz) at processing temperature 100 °C for EPDM/POE and EPDM/POE/POSS blends, applied high oscillation strain 100%. Table S1: Minimum elastic torque S’_min_, maximum elastic torque S’_max_, and the increase in elastic torque ΔS’ during 30 min at 160 °C for EPDM/POE blends; Figure S8: Cure rate dS’/dt (dNm/min) for EPDM/POE/POSS blends determined at 160 °C. Table S2: The swelling degree in toluene and sol fraction for EPDM/POE/POSS blends. Figure S9: Storage modulus G′ (kPa), loss modulus G″ (kPa) and loss factor tan δ measured using RPA rheometer after curing for 30 min at 160 °C. Tests were done at 70 °C, with an applied frequency of 1 Hz; Figure S10: Cole–Cole plots for EPDM/POE blends measured at ambient temperature in the frequency range 0.1–628 rad/s, with an applied oscillation strain of 0.05%. Table S3: The determined values for the shape-memory test: l_0_ is the initial specimen length measured at 25 °C prior to deformation, l_e_ is the length of the deformed specimen measured at 80 °C (T > T_trans_), l_f_ is the length after shape fixation and unloading at T < T_trans_, and l_r_ is the length after shape recovery upon reheating to T > T_trans_. Figure S11: Storage shear modulus G′ and loss shear modulus G″ as a function of temperature for EPDM/POE and EPDM/POE/POSS blends, with an applied frequency of 1 Hz and an applied oscillation strain of 0.05%. Table S4: Melting temperature T_m_, enthalpy of melting −ΔH_m_ and degree of crystallization χ_C_ determined based on I cycle of heating using DSC equipment. Figure S12: DSC plots for EPDM and EPDM/8137. Speed of heating/cooling, 10 °C/min. Figure S13: DSC plots for EPDM/8452 and EPDM/8180. Speed of heating/cooling, 10 °C/min. Figure S14: DSC plots for EPDM/8137/POSS and EPDM/8452/POSS. Speed of heating/cooling, 10 °C/min. Figure S15: DSC plots for EPDM/8180/POSS. Speed of heating/cooling, 10 °C/min. Figure S16: TGA plots of weight loss as a function of temperature (left) for EPDM/POE and EPDM/POE/POSS blends, and DTA plots of EPDM/POE/POSS blends (right). Figure S17: Relaxation plots. Stress as a function of time, with an applied deformation of 1%. Figure S18: Relaxation plots. Modulus G as a function of time, with an applied deformation of 1%. Table S5: Calculated based on the Maxwell model discrete relaxation spectrum. Pairs of relaxation modulus Gi, relaxation time τ_i_, and equilibrium modulus Ge. Figure S19: Creep and recovery plots for EPDM at ambient temperature, with an applied stress of 100 Pa. Figure S20: Creep compliance J as a function of time for EPDM/POE and EPDM/POE/POSS blends. Figure S21: Recovery compliance J_R_ as a function of time for EPDM/POE and EPDM/POE/POSS blends. Figure S22: Recovery strain for EPDM/POE and EPDM/POE/POSS blends. Table S6: Strain at the end of creep test ε_max_, strain at the end of recovery test ε_f_, and recovery percentage R.
