# Supplementary material for: Octavinyl-POSS as a Modifier of Thermoplastic Polyolefin Elastomers Based on Ethylene-Propylene-Diene and Ethylene-1-Octene EPDM/POEs Blends
Source: Materials (Basel). 2026 Oct 3;19(19):4216. doi: 10.3390/ma19194216 (PMC13666306; doi:10.3390/ma19194216)
Supplement: Supplementary file 1 [file materials-19-04216-s001.zip › materials-4575353-supplementary.pdf]

Supplementary materials

Octavinyl-POSS as a modifier of thermoplastic polyolefin elastomers based on ethylene-propylene-diene and ethylene-1-octene EPDM/POEs blends

Magdalena Lipińska *, Patrycja Marek and Monika Gałęziewska

Institute of Polymer and Dye Technology, Lodz University of Technology, Stefanowskiego 16, 90-537 Lodz, Poland; 247959@edu.p.lodz.pl (P.M.); monika.galeziewska@p.lodz.pl (M.G.)

* Correspondence: magdalena.lipinska@p.lodz.pl ;

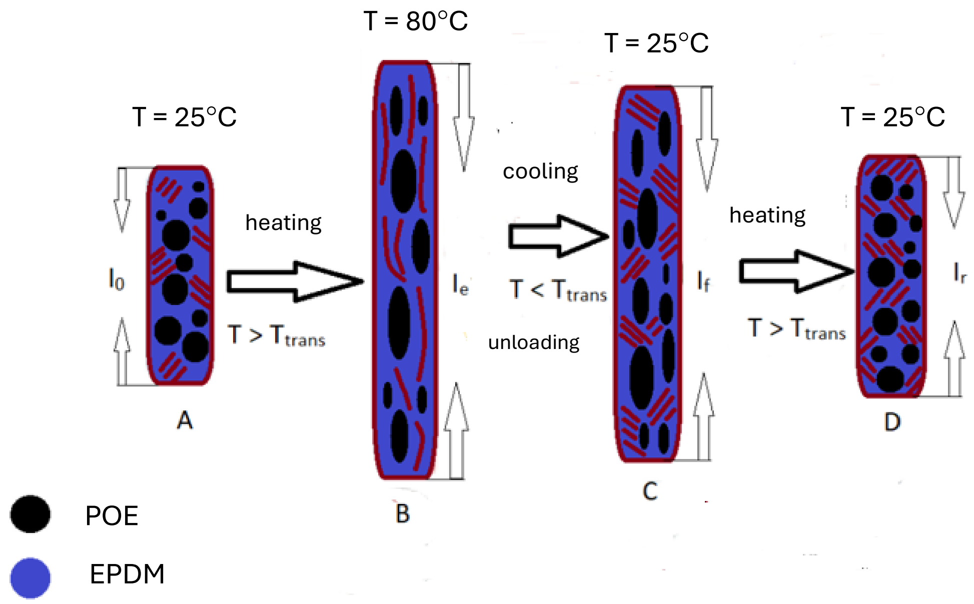

Scheme S1: The procedure applied during single shape memory test.

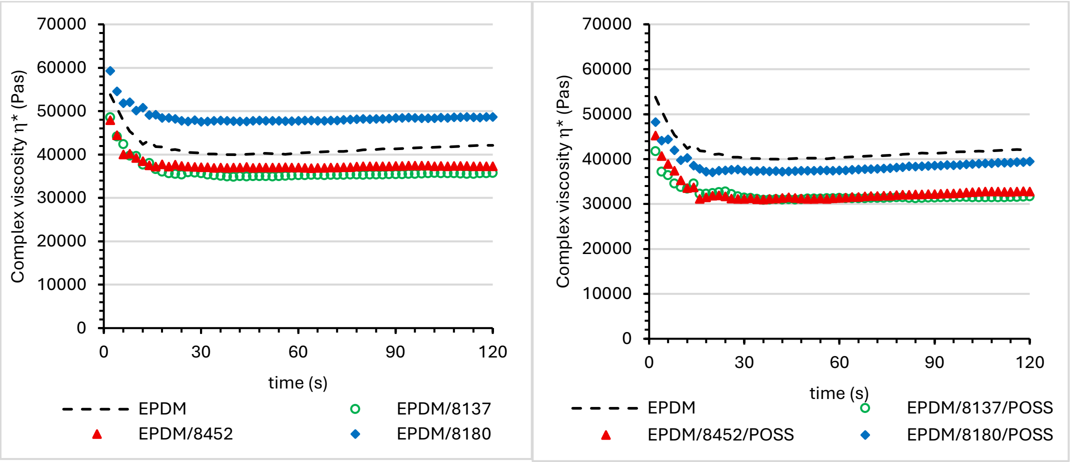

Figure S1: Complex viscosity η* (Pas) at 100°C for plasticized EPDM/POE and EPDM/POE/POSS blends.

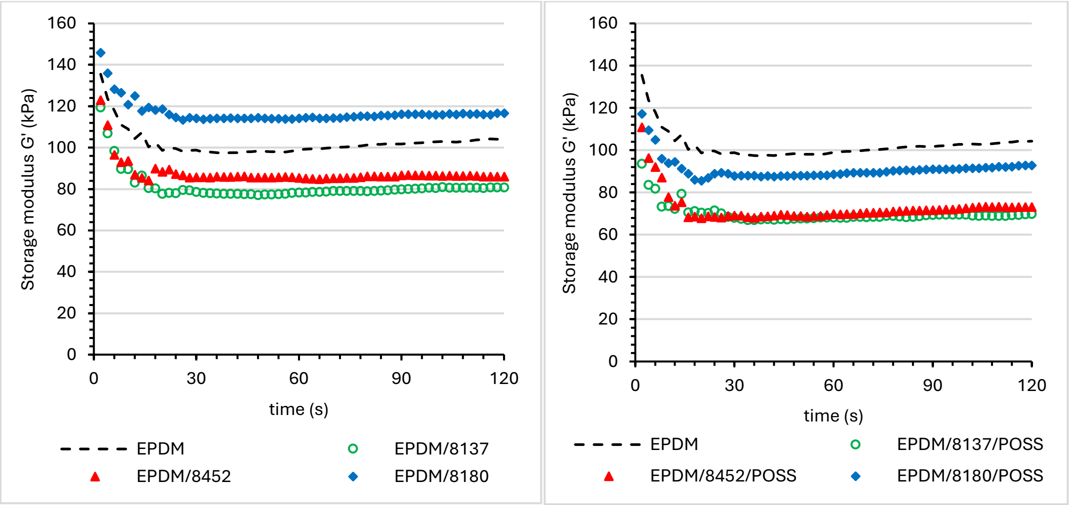

Figure S2: Storage modulus G’ (kPa) at 100°C for plasticized EPDM/POE and EPDM/POE/POSS blends.

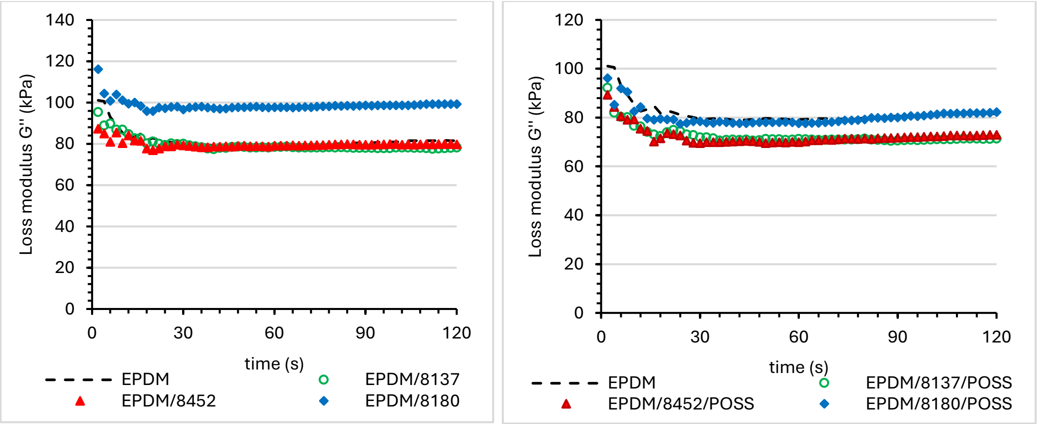

Figure S3: Loss modulus G’’ (kPa) at 100°C for plasticized EPDM/POE and EPDM/POE/POSS blends.

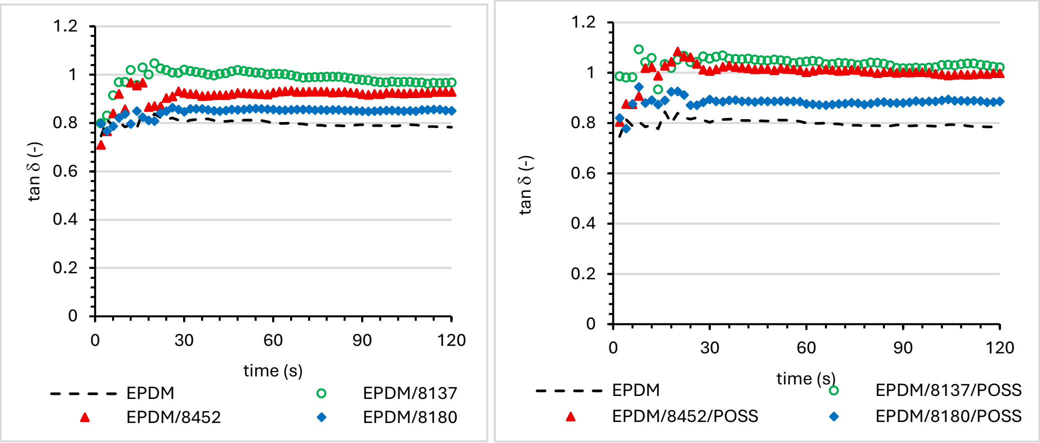

Figure S4: Loss factor tan δ (-) at 100°C for plasticized EPDM/POE and EPDM/POE/POSS blends.

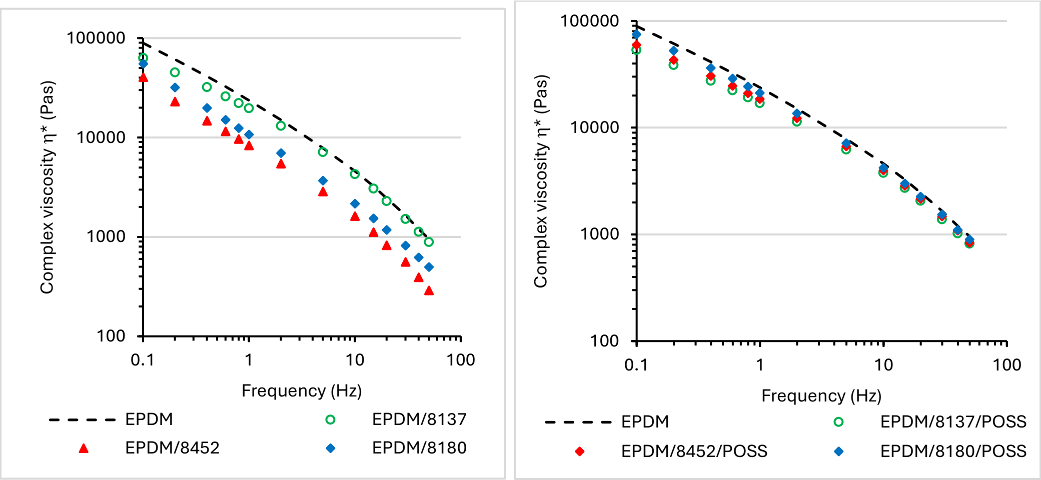

Figure S5: Complex viscosity η* as a function of applied frequency (Hz) at processing temperature 100°C for EPDM/POE and EPDM/POE/POSS blends, applied high oscillation strain 100%.

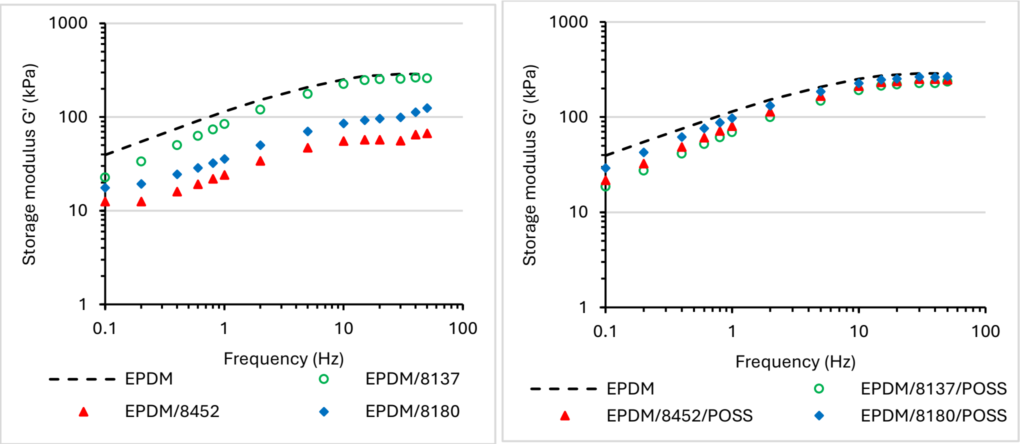

Figure S6: Storage modulus G’ (kPa) as a function of applied frequency (Hz) at processing temperature 100°C for EPDM/POE and EPDM/POE/POSS blends, applied high oscillation strain 100%.

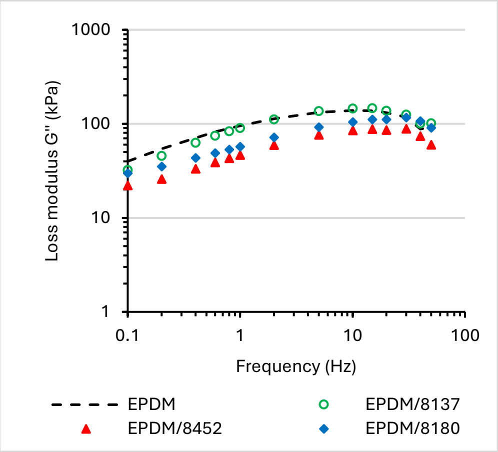

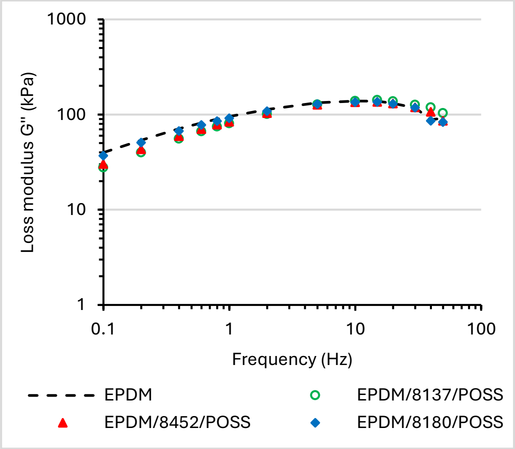

Figure S7: Loss modulus G'' (kPa) as a function of applied frequency (Hz) at processing temperature 100°C for EPDM/POE and EPDM/POE/POSS blends, applied high oscillation strain 100%.

Table S1: Minimum elastic torque S'_{min}, maximum elastic torque S'_{max}, the increase of elastic torque ΔS' during 30 min. at 160°C for EPDM/POE blends.

	S'_{min} dNm	S'_{max} dNm	ΔS' dNm
EPDM	0.92	1.04	0.11
EPDM/8137	0.58	0.66	0.08
EPDM/8452	0.68	0.77	0.09
EPDM/8180	1.02	1.05	0.03

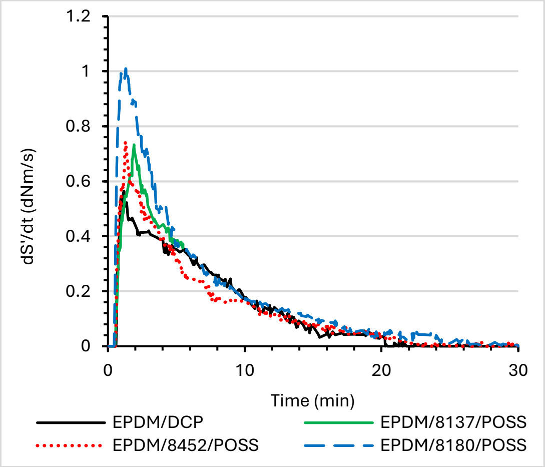

Figure S8: Cure rate dS'/dt (dNm/min.) for EPDM/POE/POSS blends determined at 160°C.

Table S2: The swelling degree in toluene and sol fraction for EPDM/POE/POSS blends

	Q_w % (*)	Sol % (**)
EPDM/DCP	615±38	8.8±0.7
EPDM/8137/POSS	478±12	8.6±1.1
EPDM/8452/POSS	480±18	8.6±0.8
EPDM/8180/POSS	503±5	8.0±0.5

*Q_w equlibrum swelling calculated according to the equation:
$Q_w = \frac{m_{sw} - m_s}{m_s}$ x 100, where: m_{sw} - mass of the sample swollen in toluene for 48 h (mg), m_s - mass of the sample dried after swelling at 50 °C during 24 h (mg)
** Sol calculated according to the equation:
$Sol = \frac{m_0 - m_s}{m_0}$ x 100 , where m_0 – mass of the sample before swelling, m_s - mass of the sample dried after swelling at 50 °C during 24 h (mg)

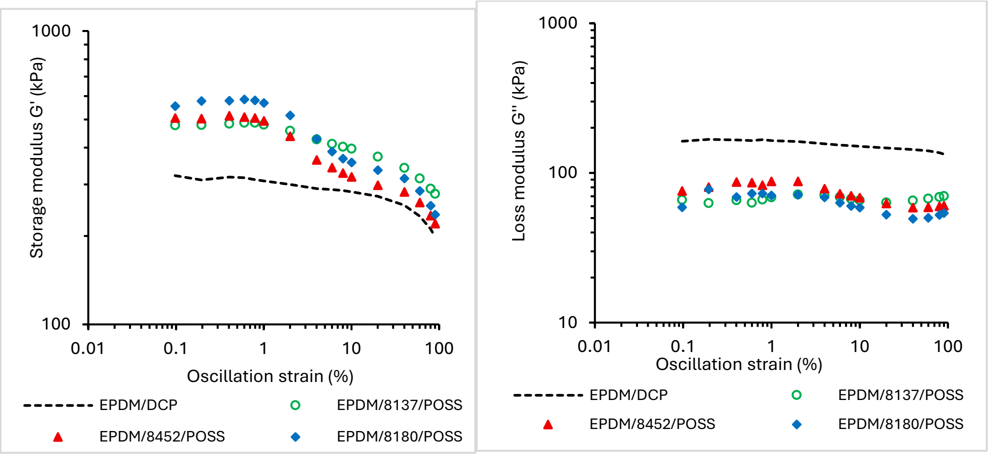

Figure S9: Storage modulus G' (kPa), loss modulus G'' (kPa) and loss factor tan δ measured using RPA rheometer after curing during 30 min at 160°C. Test done at 70°C, applied frequency 1Hz.

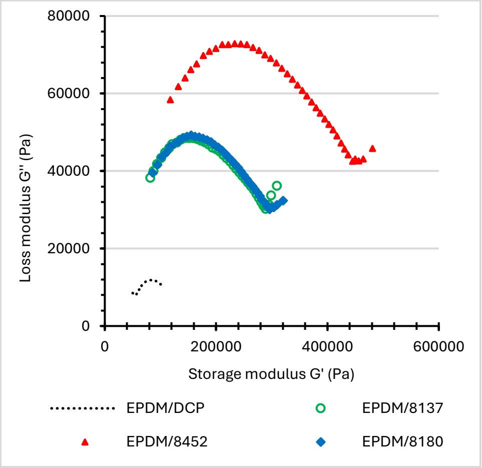

Figure S10: Cole-Cole plots for EPDM/POE blends measured at ambient temperature in frequency range 0.1-628 rad/s, applied oscillation strain 0.05%.

Table S3: The determined for shape-memory test values, l_0 is the initial specimen length measured at 25°C prior to deformation, l_e is the length of the deformed specimen measured at 80°C ($T > T_{trans}$), l_f is the length after shape fixation and unloading at $T < T_{trans}$, and l_r is the length after shape recovery upon reheating to $T>T_{trans}$.

	l_0 (mm)	l_e (mm)	l_f (mm)	l_r (mm)
EPDM/8137	17.19	27.89	23.33	21.93
EPDM/8137	21.58	32.89	29.85	26.55
EPDM/8137	19.24	29.45	26.35	23.98
EPDM/8452	17.42	28.85	24.11	22.77
EPDM/8452	24.40	36.88	32.61	29.99
EPDM/8452	22.11	31.29	28.32	26.12
EPDM/8180	17.85	28.95	24.68	22.22
EPDM/8180	22.83	33.75	29.99	27.92
EPDM/8180	21.68	32.48	28.78	26.87
EPDM/8137/POSS	14.93	25.82	24.38	15.86
EPDM/8137/POSS	15.78	26.83	25.45	16.02
EPDM/8137/POSS	14.68	22.08	21.72	15.10
EPDM/8452/POSS	15.35	26.77	26.73	15.7
EPDM/8452/POSS	15.23	24.03	23.34	15.92
EPDM/8452/POSS	13.69	18.58	18.13	13.70
EPDM/8180/POSS	15.08	25.36	25.24	15.18
EPDM/8180/POSS	15.46	23.01	22.61	15.65
EPDM/8180/POSS	15.35	22.77	22.25	15.88

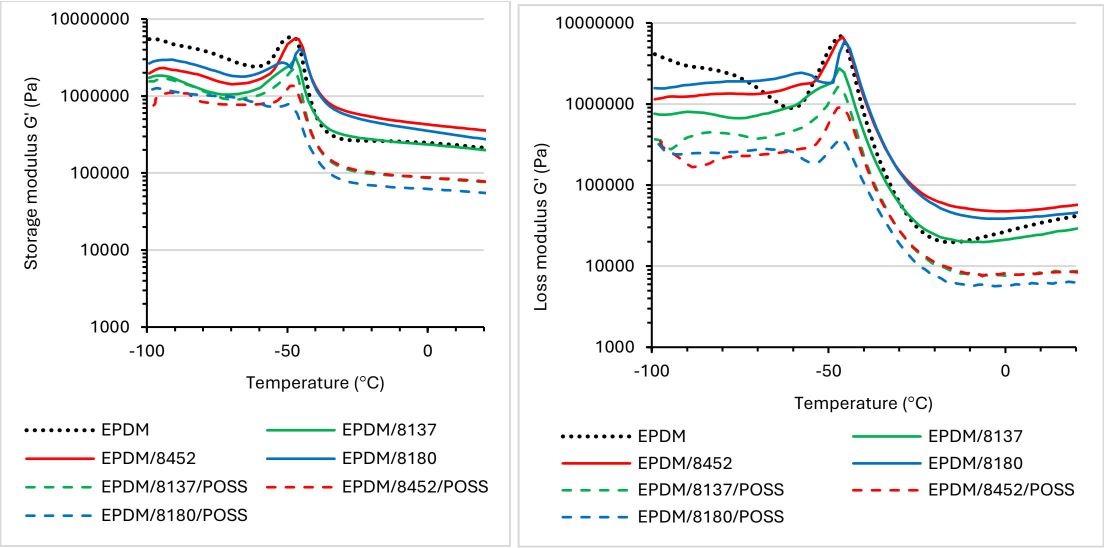

Figure S11: Storage shear modulus G' and loss shear modulus G'' as a function temperature for EPDM/POE and EPDM/POE/POSS blends. Applied frequency 1Hz, applied oscillation strain 0.05%.

Table S4: Melting temperature T_m, enthalpy of melting $-\Delta H_m$ and degree of crystallization χ_C determined based on I cycle of heating using DSC equipement.

Sample	T_m (°C) *	$-\Delta H_m$ (mJ/g) *	χ_c (I cycle)
EPDM	51.0	4.22	1.46
EPDM/8137	52.2	5.39	9.29
EPDM/8452	74.2	8.37	14.43
EPDM/8180	49.8	4.75	8.19
EPDM/8137/POSS	42.4	2.91	5.02
EPDM/8452/POSS	71.2	3.35	5.78
EPDM/8180/POSS	42.7	1.37	2.36

* first cycle of sample heating

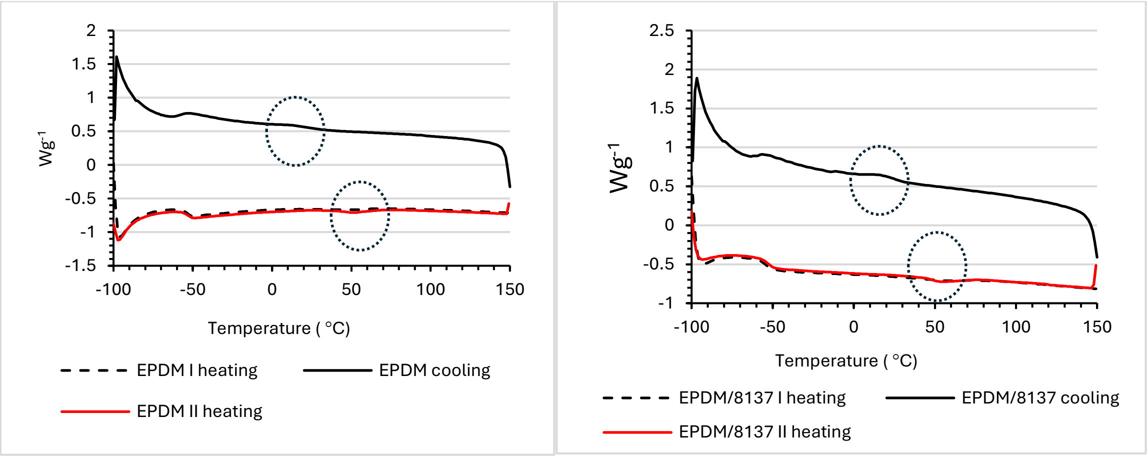

Figure S12: DSC plots for EPDM and EPDM/8137. Speed of heating/cooling 10°C/min.

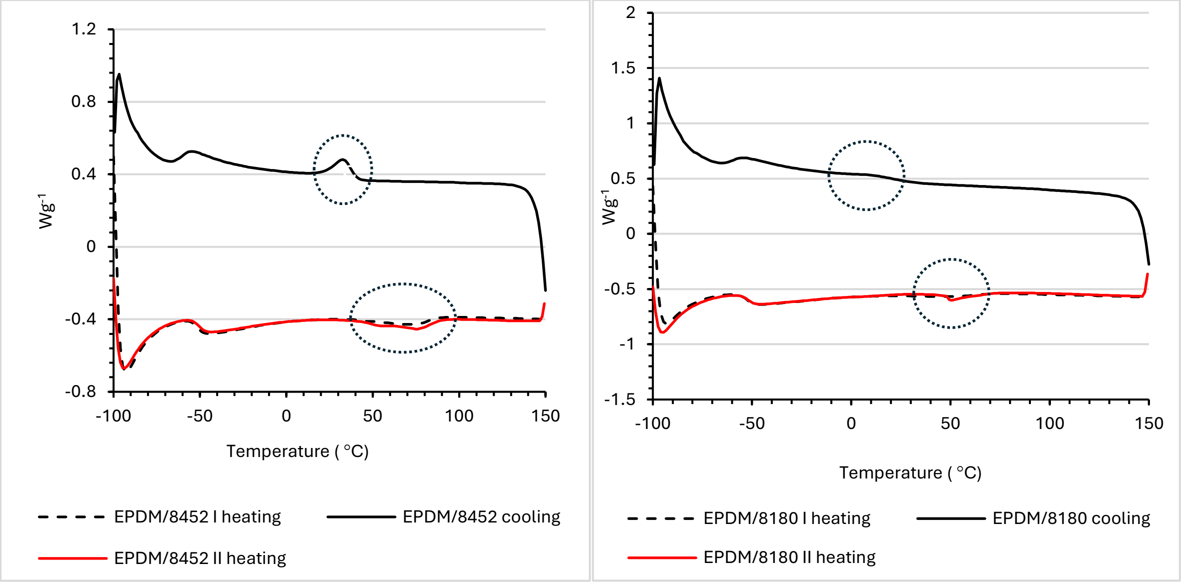

Figure S13: DSC plots for EPDM/8452 and EPDM/8180. Speed of heating/cooling 10°C/min.

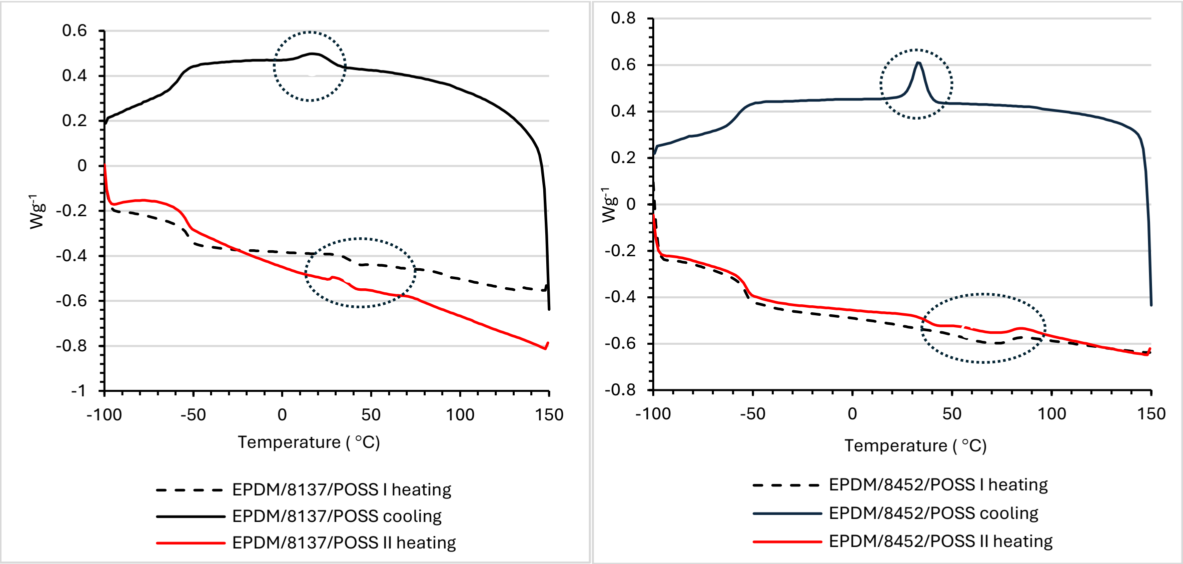

Figure S14: DSC plots for EPDM/8137/POSS and EPDM/8452/POSS. Speed of heating/cooling 10°C/min.

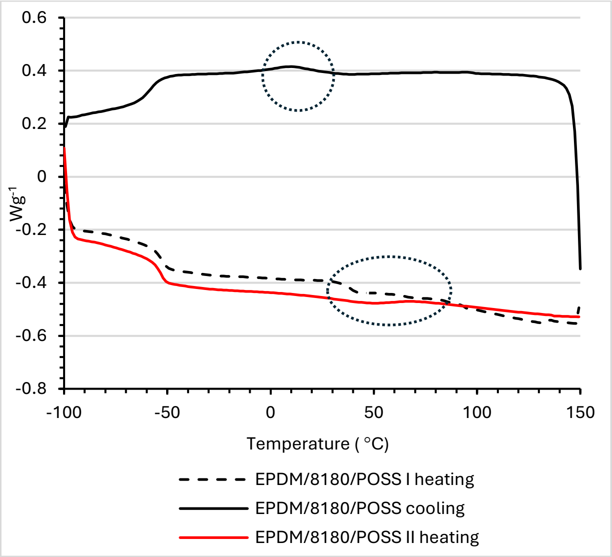

Figure S15: DSC plots for EPDM/8180/POSS. Speed of heating/cooling 10°C/min.

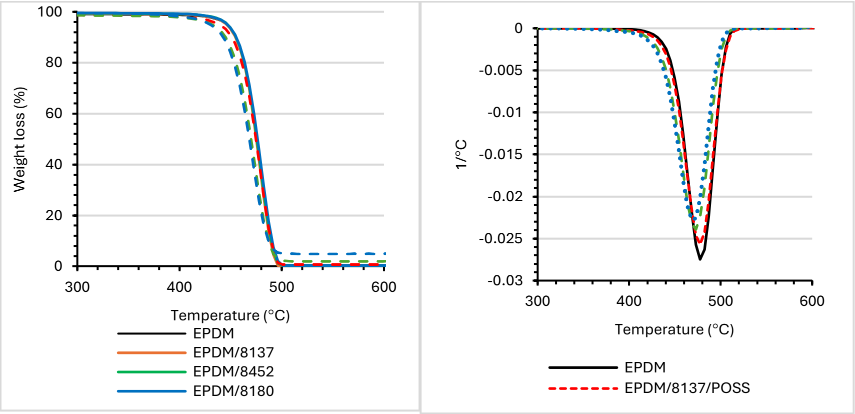

Figure S16: TGA plots of weight loss as a function of temperature (left) for EPDM/POE and EPDM/POE/POSS blends, DTA plots of EPDM/POE/POSS blends (right).

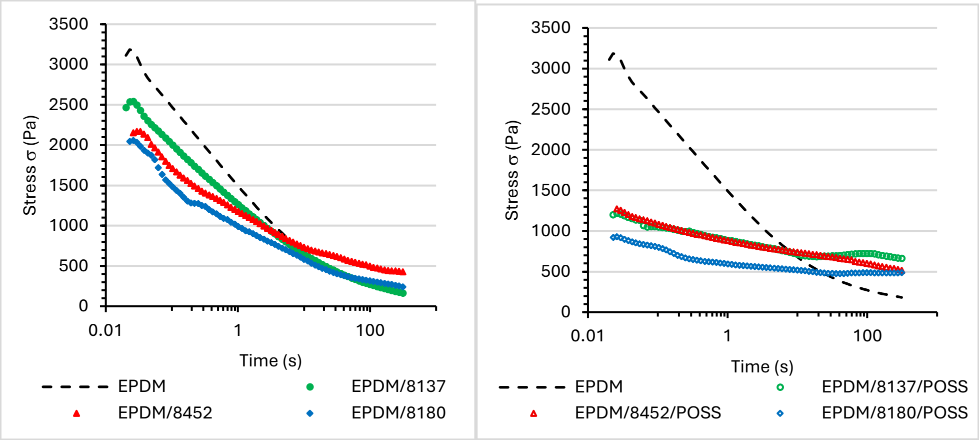

Figure S17: Relaxation plots. Stress as a function of time, applied deformation 1%.

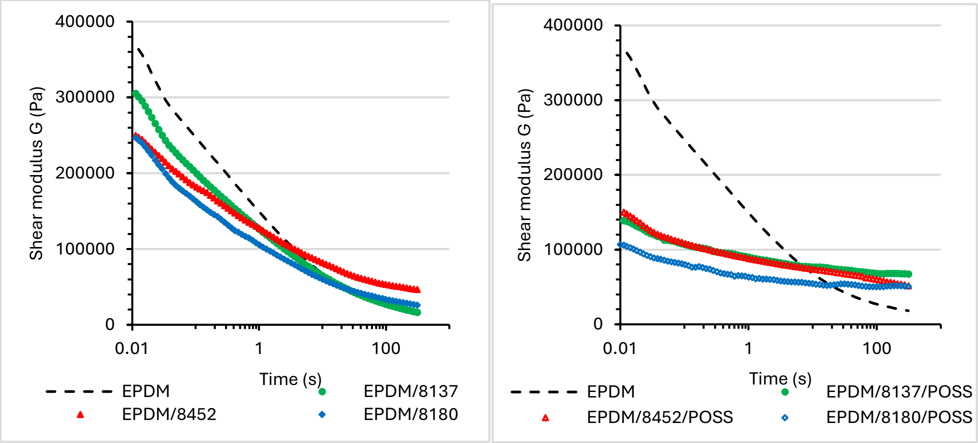

Figure S18: Relaxation plots. Modulus G as a function of time, applied deformation 1%.

Table S5: Calculated based on Maxwell model discrete relaxation spectrum. Pairs of relaxation modulus G_i and relaxation time τ_I, equilibrium modulus G_e.

	G_e (Pa)	G_i (Pa)	τ_i (s)	R^2
EPDM	20052,1	171945<br>121540<br>49979,9	0,100563<br>2,84495<br>45,5708	0,994
EPDM/8137	18242,3	144986<br>92623<br>46939,6	0,088975<br>3,05918<br>57,0135	0,993
EPDM/8452	49514,1	91327,5<br>68153,1<br>44528,6	0,0756312<br>1,71706<br>31,6161	0,997
EPDM/8180	28047,9	117992<br>70065,4<br>35491,2	0,075263<br>2,54839<br>47,0107	0,995
EPDM/8137/POSS	67831,7	45380,6<br>23592,7<br>13466,2	0,034334<br>1,07638<br>31,235	0,998
EPDM/8452/POSS	52737,1	60313.9<br>27693.4<br>24824.2	0,032103<br>1,16876<br>73,4411	0,997
EPDM/8180/POSS	51459,9	30872,7<br>22442,9<br>11392,3	0,022755<br>0,329033<br>6,99564	0,996

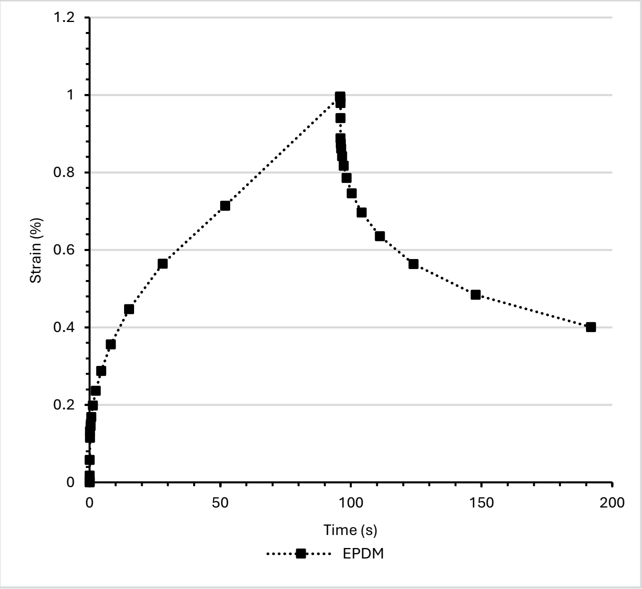

Figure S19: Creep and recovery plots for EPDM at ambient temperature, applied stress 100Pa.

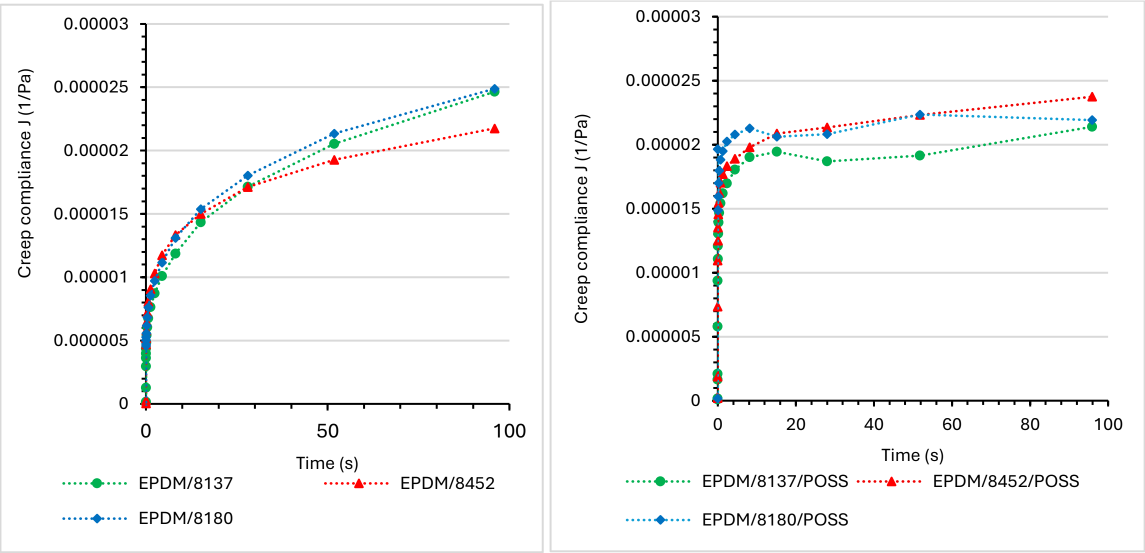

Figure S20: Creep compliance J as a function of time for EPDM/POE and EPDM/POE/POSS blends.

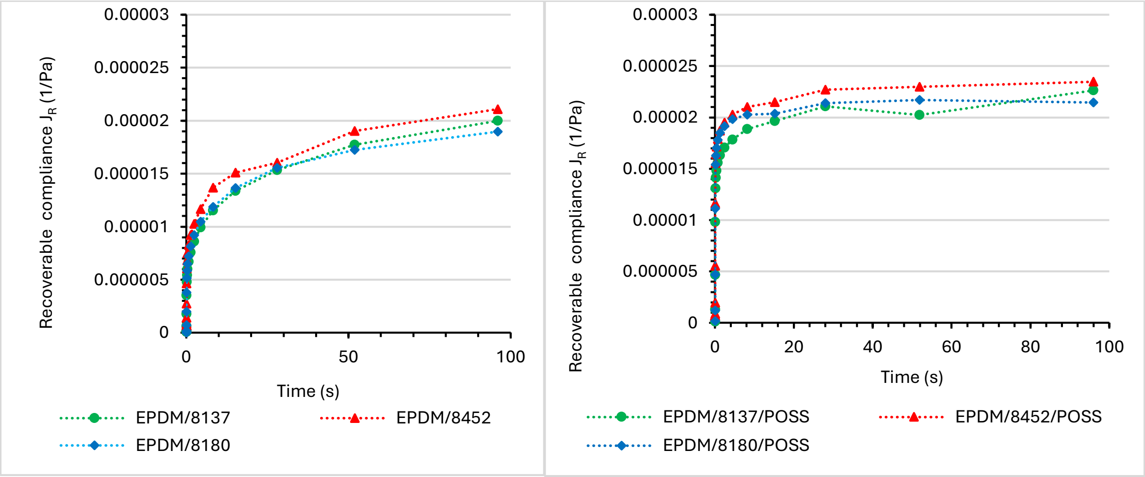

Figure S21: Recovery compliance J_R as a function of time for EPDM/POE and EPDM/POE/POSS blends.

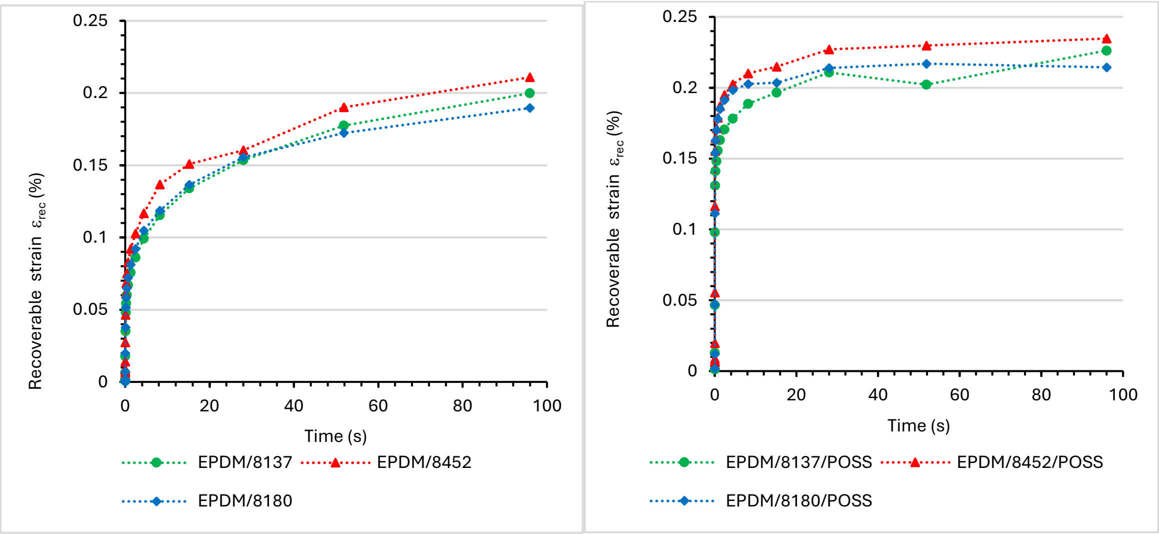

Figure S22: Recovery strain for EPDM/POE and EPDM/POE/POSS blends.

Table S6: Strain at the end of creep test ε_{max}, strain at the end of recovery test ε_f, recovery percentage R.

	ε_{MAX} %	ε_F %	R % (*)
Uncured EPDM	0.901166	0.400618	55.5
EPDM/8137	0.246485	0.072645	70.5
EPDM/8452	0.217612	0.023058	89.4
EPDM/8180	0.248903	0.07817	68.6
EPDM/8137/POSS	0.213995	0.0046663	97.8
EPDM/8452/POSS	0.237504	0.0090454	96.2
EPDM/8180/POSS	0.219255	0.000437758	99.8

(*) Recovery percentage was calculated according to the equation:

$$R = \frac{\varepsilon_{max} - \varepsilon_f}{\varepsilon_{max}} \times 100\%$$
